# Classification models using circulating neutrophil transcripts can detect unruptured intracranial aneurysm

**DOI:** 10.1186/s12967-020-02550-2

**Published:** 2020-10-15

**Authors:** Kerry E. Poppenberg, Vincent M. Tutino, Lu Li, Muhammad Waqas, Armond June, Lee Chaves, Kaiyu Jiang, James N. Jarvis, Yijun Sun, Kenneth V. Snyder, Elad I. Levy, Adnan H. Siddiqui, John Kolega, Hui Meng

**Affiliations:** 1 Canon Stroke and Vascular Research Center, Clinical and Translational Research Center, 875 Ellicott Street, Buffalo, NY 14214 USA; 2 Department of Biomedical Engineering, University of Buffalo, Buffalo, USA; 3 Department of Computer Science and Engineering, University of Buffalo, Buffalo, USA; 4 Department of Neurosurgery, Jacobs School of Medicine and Biomedical Sciences, Buffalo, USA; 5 Genetics, Genomics, and Bioinformatics Program, Jacobs School of Medicine and Biomedical Sciences, Buffalo, USA; 6 Department of Pediatrics, Jacobs School of Medicine and Biomedical Sciences, Buffalo, USA; 7 Department of Microbiology and Immunology, Jacobs School of Medicine and Biomedical Sciences, Buffalo, USA; 8 Department of Radiology, Jacobs School of Medicine and Biomedical Sciences, Buffalo, USA; 9 Department of Neurology, Jacobs School of Medicine and Biomedical Sciences, Buffalo, USA; 10 Department of Pathology and Anatomical Sciences, Jacobs School of Medicine and Biomedical Sciences, Buffalo, USA; 11 Department of Mechanical & Aerospace Engineering, University At Buffalo, Buffalo, NY USA; 12 Department of Internal Medicine, Jacobs School of Medicine and Biomedical Sciences, Buffalo, USA

**Keywords:** Intracranial aneurysm, Neutrophil, Transcriptomics, Machine learning, Inflammation, Prediction model

## Abstract

**Background:**

Intracranial aneurysms (IAs) are dangerous because of their potential to rupture. We previously found significant RNA expression differences in circulating neutrophils between patients with and without unruptured IAs and trained machine learning models to predict presence of IA using 40 neutrophil transcriptomes. Here, we aim to develop a predictive model for unruptured IA using neutrophil transcriptomes from a larger population and more robust machine learning methods.

**Methods:**

Neutrophil RNA extracted from the blood of 134 patients (55 with IA, 79 IA-free controls) was subjected to next-generation RNA sequencing. In a randomly-selected training cohort (n = 94), the Least Absolute Shrinkage and Selection Operator (LASSO) selected transcripts, from which we constructed prediction models via 4 well-established supervised machine-learning algorithms (K-Nearest Neighbors, Random Forest, and Support Vector Machines with Gaussian and cubic kernels). We tested the models in the remaining samples (n = 40) and assessed model performance by receiver-operating-characteristic (ROC) curves. Real-time quantitative polymerase chain reaction (RT-qPCR) of 9 IA-associated genes was used to verify gene expression in a subset of 49 neutrophil RNA samples. We also examined the potential influence of demographics and comorbidities on model prediction.

**Results:**

Feature selection using LASSO in the training cohort identified 37 IA-associated transcripts. Models trained using these transcripts had a maximum accuracy of 90% in the testing cohort. The testing performance across all methods had an average area under ROC curve (AUC) = 0.97, an improvement over our previous models. The Random Forest model performed best across both training and testing cohorts. RT-qPCR confirmed expression differences in 7 of 9 genes tested. Gene ontology and IPA network analyses performed on the 37 model genes reflected dysregulated inflammation, cell signaling, and apoptosis processes. In our data, demographics and comorbidities did not affect model performance.

**Conclusions:**

We improved upon our previous IA prediction models based on circulating neutrophil transcriptomes by increasing sample size and by implementing LASSO and more robust machine learning methods. Future studies are needed to validate these models in larger cohorts and further investigate effect of covariates.

## Background

Intracranial aneurysms (IAs) are present in up to 6% of the general population, but only about 10% show any symptoms prior to rupture [1]. The rupture of an IA is the leading cause of nontraumatic subarachnoid hemorrhage, which has a mortality rate up to 50% [2–4]. Clinical studies have shown that the incidence of future rupture can be decreased with elective endovascular or surgical treatment [5, 6]. However, because most IAs are asymptomatic, unruptured aneurysms are usually only detected incidentally on cerebral imaging performed for other medical reasons. Imaging by magnetic resonance imaging/angiography (MRI/MRA), computed tomography angiography (CTA), and digital subtraction angiography (DSA) are not routinely used for IA screening because they are expensive and carry the risk for serious complications due to their invasive nature (e.g., DSA) or exposure to radiation (e.g., DSA, CTA). Thus, a blood test to identify individuals with unruptured IAs could facilitate a paradigm shift to proactive IA management by enabling routine screening and preventive treatment.

We hypothesized that changes in circulating neutrophil gene expression are correlated with the presence of IA in the cerebral vasculature. Neutrophils, the most abundant leukocyte in human blood, are important sentinels for tissue damage and defense against microorganisms, and play a central role in the coordination of innate immunity and inflammation [7]. Recent evidence has suggested that neutrophils have a large degree of heterogeneity in both phenotype and function, playing a wide range of roles in homeostasis and disease-specific responses [8]. Indeed, in previous publications, our group has demonstrated different circulating neutrophil signatures between individuals with cystic fibrosis and idiopathic arthritis [7], suggesting that neutrophils have the ability to adapt their transcriptomes to specific biologic contexts.

Recent studies have begun to show that neutrophils may display disease-specific responses during IA pathophysiology. Investigations of resected aneurysms have found elevated levels of key proteins released from neutrophils, namely myeloperoxidase (*MPO*) and neutrophil gelatinase-associated lipocalin (*NGAL*) in the IA wall [9, 10]. These proteins have also been found to be elevated in plasma levels of blood from patients with IA [10, 11]. These proteins may play a key role in extracellular matrix (ECM) degradation and neutrophil activation [12, 13] during aneurysm natural history via their production of reactive oxygen species (ROS) and protection of MMP-9 degradation, respectively [9, 10]. Neutrophils can produce a wide range of cytokines, namely inflammatory and immunoregulatory cytokines, chemokines (necessary for monocyte infiltration), angiogenic and fibrogenic factors, and tumor necrosis factor (TNF) superfamily members [14]. Studies have shown that TNF is upregulated in human IA tissues [15], and from animal models of the disease, is essential for IA formation [16, 17]. Additionally, based on their reported role in progression and rupture of abdominal aortic aneurysms, neutrophils may also release neutrophil extracellular traps (NETs) in IA [18–21].

In a small, proof-of-concept study, we previously performed differential expression analysis in case-controlled cohorts (n = 11 IA, n = 11 control) and found an 82-gene signature that distinguishes IAs from controls [22]. Bioinformatics analyses broadly reflected peripheral neutrophil activation in patients with IA, as genes with elevated expression in the IA group were associated with leukocyte activation, cell activation, and defense response [22]. In a follow-up study, we next utilized machine learning to determine whether an algorithm could predict the presence of unruptured IA using differential gene expression [23]. In an unmatched cohort (n = 30), 26 highly-informative neutrophil transcripts (FDR < 0.05, abs[fold-change] ≥ 1.5) were used to construct a diagonal Linear Discriminant Analysis model, which predicted the presence of IA with an accuracy of 90% in a small independent cohort (n = 10) [23].

While these results were exciting, due to the small sample size, it was difficult to generalize our findings to a broader population. Therefore, in this study, we aimed at confirming these results in a larger cohort of patients. Importantly, this increased sample size would enable us to: (A) implement more advanced feature selection methods (in place of basic thresholding) and machine learning techniques (i.e. Random Forest) to improve prediction accuracy, and (B) examine potential effect of demographics and comorbidities on model prediction.

## Methods

### Study enrollment

This study was approved by University at Buffalo Institutional Review Board (study no. 030-474433). All methods followed the approved protocol. Written informed consent was obtained from all subjects prior to sample collection. Patients receiving cerebral DSA at Gates Vascular Institute, Buffalo, NY with and without IA diagnosis were enrolled in this study. Most indications for DSA included confirmation of noninvasive imaging results of presence of IAs or other cerebral vascular conditions, follow-up of non-invasive imaging for headache or visual disturbance, or follow-up of previously identified IAs. All patients who consented to participate in this study were over 18 years, English speaking, and had not previously been treated for IA. Patients with potentially altered immune systems were excluded, including, for example, patients who had recent invasive surgery, were receiving chemotherapy, had a fever (> 100°F), had received solid organ transplants, had autoimmune diseases, or were taking prednisone or other immunomodulating drugs.

Between December 2013 and September 2018, we collected 232 blood samples from cerebral DSA patients at Gates Vascular Institute (103 from patients with IA, and 129 from IA-free controls). Forty-three of these samples had been sequenced as a part of our previous studies [22, 23]. In all cases, IA diagnosis was confirmed by DSA images. Patient medical record data was also collected to study demographics and comorbidities.

### Neutrophil isolation

During DSA, 16 mL of blood was drawn from the access catheter in the femoral artery and transferred into two 8 mL, citrated, cell preparation tubes (BD, Franklin Lakes, NJ). Neutrophils were isolated within 1 h of blood collection, as described elsewhere [7]. Briefly, cell preparation tubes were centrifuged at 1700×*g* for 25 min to separate erythrocytes and neutrophils from mononuclear cells and plasma in the peripheral blood samples via a Ficoll density gradient. Erythrocytes and neutrophils were collected into a 3 mL syringe. Following hypotonic lysis of red blood cells, neutrophils were isolated by centrifugation at 400×*g* for 10 min, disrupted and stored in TRIzol reagent (Life Technologies, Carlsbad, CA) at − 80 °C until further processing. Neutrophils isolated in this fashion are more than 98% CD66b+ by flow cytometry and contain no contaminating CD14+ monocytes [24].

### RNA preparation

Neutrophil RNA was extracted as described previously [22] using TRIzol, according to the manufacturer’s instructions. Trace DNA was removed by DNase I (Life Technologies, Carlsbad, CA) treatment. RNA was purified using the RNeasy MinElute Cleanup Kit (Qiagen, Venlo, Limburg, Netherlands) and suspended in RNase-free water. The purity and concentration of RNA in each sample were measured by absorbance at 260 nm and 280 nm on a NanoDrop 2000 spectrophotometer (Thermo Scientific, Waltham, MA), and 200 ng to 400 ng of RNA was reserved for sequencing. Precise RNA concentration was measured via the Quant-iT RiboGreen Assay (Invitrogen, Carlsbad, CA) with a TBS-380 Fluorometer (Promega, Madison, WI), and the quality of the RNA samples was measured with an Agilent 2100 BioAnalyzer RNA 6000 Pico Chip (Agilent, Las Vegas, NV). RNA samples to be sequenced had acceptable purity (260/280 ratio of ~ 1.8 or greater, range: 1.76–2.12) and integrity (RIN of ~ 5 or greater, range: 4.5–9.1) prior to RNA sequencing.

### RNA sequencing

For newly processed samples, the Illumina TruSeq Stranded Total RNA Gold Library Preparation Kit (Illumina, San Diego, CA) was used for library preparation. Samples were subjected to 50-cycle, single-read sequencing in a HiSeq2500 system (Illumina) and demultiplexed using Bcl2Fastq. To increase sample size, we combined reads from these new samples with reads from our previous samples [22, 23] that were sequenced in the same manner, but for which libraries were constructed using the Illumina TruSeq RNA library Prep Kit V2 (Illumina, San Diego, CA). For all data, per-cycle base-call (BCL) files generated by the Illumina HiSeq2500 were converted to per-read FASTQ files using bcl2fastq version 2.20.0.422 using default parameters. The quality of the sequencing was reviewed using FastQC version 0.11.5. Detection of potential contamination was done using FastQ Screen version 0.11.1. FastQC and FastQ Screen quality reports were summarized using MultiQC version 1.5. No adapter sequences were detected, so no trimming was performed. Genomic alignments were performed using HISAT2 version 2.1.0 using default parameters. NCBI reference GRCh38 was used for the reference genome and gene annotation set. Sequence alignments were compressed and sorted into binary alignment map (BAM) files using samtools version 1.3. Counting of mapped reads for genomic features was performed using Subread featureCounts version 1.6.2 using the parameters -s 2 –g gene_id –t exon –Q 60, the annotation file specified with—a was the NCBI GRCh38 reference from Illumina iGenomes. Aggregate quality control data (i.e. alignment statistics and feature assignment statistics) were again summarized using MultiQC.

### Differential expression analysis and data exploration

Before implementing our machine learning pipeline, we performed differential expression analysis on the whole dataset to identify transcripts that were significantly differentially expressed in IA using Bioconductor package edgeR version 3.24.0. After estimating dispersion, edgeR identified differentially expressed genes by using a negative binomial distribution with generalized linear models and a quasi-likelihood F-test to identify differentially expressed genes [25, 26]. We incorporated the two sequencing batches into the design matrix to correct for any potential batch effects due to different library preparation kits. Genes with a counts sum > 0 across all samples were used as input. Multiple hypothesis testing correction was performed using Benjamini–Hochberg false discovery rate (FDR) correction [27]. Transcripts with an FDR-corrected p-value (q-value) < 0.05 were considered significantly differentially expressed. To explore how transcriptomes separated patients with and without IA on a broad scale, we performed hierarchical clustering, using the hclust package in R under default settings (complete linkage).

Next-generation sequencing is typically performed on high quality RNA (RIN > 7), when possible [28]. However, clinical samples, particularly human neutrophils that contain high levels of endonucleases as part of the host defense response to bacteria, rarely produce RNA without any degree of degradation [29]. Between IA and control groups in our study, there was no statistically significant difference in RIN (p = 0.18, Student’s t-test). Yet, given that some samples had low RIN and others had high RIN, we performed co-variate correlation analysis as shown in Xiong et al. [30] in order to determine if RNA quality could have affected the expression levels of differentially expressed transcripts. A correlation between gene expression and RIN was considered if it had both a Pearson correlation coefficient r > 0.80 and a p-value < 0.01.

### Verification of expression differences by qPCR in a sub-cohort

To verify expression differences in differentially expressed genes, quantitative polymerase chain reaction (qPCR) was performed. Due to limitations in RNA quantity, qPCR was performed on 10 transcripts in a subset of 50 of the 134 samples (20 IA and 30 control). We followed the protocol described previously [22]. In brief, oligonucleotide primers were designed using Primer3 software (Primer3Web 0.4.0) and Primer BLAST (NCBI, Bethesda, MD) to have a 60 °C melting temperature, a length of 15–25 nucleotides, and a product of 50–250 base pairs (with at least one primer that spans an exon-exon junction), as well as an estimated efficiency > 0.8 (actual range: 0.82–1.1, average: 0.96, median: 1.0) [31]. Primer sequences, annealing temperatures, efficiencies, and product lengths are reported in Additional file 1: Table S1. For reverse transcription, first-strand cDNA was generated from total RNA using qScript cDNA Synthesis kit (Quantabio, Beverly, MA, USA) according to the manufacturer’s directions. qPCR was run with 5 ng of cDNA in 20 µL reactions in triplicate in Bio-Rad CFX Connect (Bio-Rad, Hercules, California) using the qScript One-Step SYBR Green Master Mix kit (Quantabio, Beverly, MA, USA) and gene-specific primers at a concentration of 0.02 μM each. The temperature profile consisted of an initial step of 95 °C for 1 min, followed by 40 cycles of 95 °C for 15 s and 60 °C for 1 min, and then a final melting curve analysis from 60 to 95 °C over 20 min.

Gene-specific amplification was demonstrated by a single peak using the Bio-Rad dissociation melt curve. Samples were normalized based on *HPRT1, GAPDH,* and *GPI* (housekeeping genes [32–34]) expression, which were run in parallel reactions to the genes of interest. Additional file 2: Fig. S1 shows the stability in the expression (from RNA sequencing, Additional file 2: Fig. S1A) and C_t_ values (from qPCR, Additional file 2: Fig. S1B) of the housekeeping genes in the control and IA groups. All had similar coefficient of variation values, and there was no statistical significance in their variation between the two groups for sequencing or qPCR (all p-values > 0.01, F-test). Their C_t_ values were used to calculate gene-specific average fold-change between the two groups using the 2^−ΔΔCt^ method [35]. These values were then averaged across all housekeeping genes. For comparison with qPCR data, RNA sequencing data from the same samples was used to calculate the average fold-change in gene expression between the IA and the control groups. This fold-change value (presented as an absolute fold-change in the positive or negative direction [for fold-change < 1]) was then compared to the same metric calculated by the 2^−ΔΔCt^ method in qPCR data in order to determine if the absolute fold-change in expression was in the same direction and statistically different (Student’s t-test, significance at p-value < 0.05).

### Feature selection for classification model development

To build predictive models for IA, we began with raw counts to eliminate bias and uncertainty associated with distribution modeling incorporated in edgeR. Raw counts were then normalized to transcript per million (TPM) values to facilitate comparison of expression between samples by normalizing by both gene length and sequencing depth. Then, we applied an abundance filtering by only selecting protein coding genes with average TPM > 1 across all samples, reducing the set of potential transcripts to 18,833. To account for the two sequencing batches in our study design (as done in edgeR analyses), we performed batch effect correction using ComBat under the default settings in R [36, 37]. Then, 70% of samples were randomly allocated to a training cohort (n = 39 IA and n = 55 control) and 30% to a testing cohort (n = 16 IA and n = 24 control), maintaining the proportion of IA and controls.

For feature selection in the training cohort, we performed a supervised feature selection by using the Hilbert–Schmidt Independence Criterion Least Absolute Shrinkage and Selection Operator (HSIC LASSO) method. HSIC LASSO was implemented in the ComBat corrected dataset to select features for the model. To visualize how those selected transcripts separated samples from patients with and without IAs, we performed principal component analysis (PCA) using the prcomp package under the default settings [38]. Co-variate correlation analysis following Xiong et al. [30] was performed again to determine if RNA quality (i.e. RIN) could have affected the expression levels of TPM-normalized transcripts selected by LASSO. A correlation between gene expression and RIN was considered if it had both a Pearson correlation coefficient r > 0.80 and a p-value < 0.01.

### Model training

We used MATLAB Statistics and Machine Learning Toolbox (MathWorks, Natick, MA) to train 4 popular algorithms (K-Nearest Neighbor, Random Forest, Support Vector Machine with Gaussian and cubic kernels) on 2 different gene panels—the 37 transcripts identified by LASSO in this study and the 26 transcripts identified by filtering in our previous study. While these algorithms have been used in other disease classification applications [39–44], we implemented all 4 algorithms to determine which best suited our data. Specific parameters for each algorithm are as follows:

- For K-Nearest Neighbors, we used a Euclidean metric and 10 neighbors (k). The resulting model classified test samples by calculating their distance to each training sample and the test sample labels were predicted by choosing the class that was most common among their 10 nearest neighbors.
- For Random Forest, which constructs a multitude of decision trees in training and outputs the mode of the classes as the predicted class [45], we set the number of trees as 1000. The Random Forest was built by constructing a multitude of decision trees based on subsets of the training data generated by random sampling with replacement and the resulting model classified testing samples by the majority vote of the decision trees.
- For Support Vector Machines, we used two different kernels [46], Gaussian and cubic. To separate a binary-labeled sample, the Support Vector Machine transforms them into a multidimensional space using the kernel, and then a hyper-plane, which maximizes the distance to samples of either class, is established. The resulting model classified testing samples by transforming them into a higher dimensional space with the corresponding kernel and making decisions based on their signed distance to the hyper-plane.

### Model assessment

We estimated the performance of each model for the new LASSO-identified features as well as our previously-identified 26 features by a leave-one-out (LOO) cross-validation within the training cohort. Model predictions were compared to each patient’s clinical diagnosis from imaging, and the true positives, true negatives, false positives, and false negatives were tallied. We then calculated each model’s sensitivity, specificity, and accuracy, as described elsewhere [23]. Based on model predictions, we created receiver operating characteristic (ROC) curves and calculated the area under the ROC curve (AUC) to assess model performance. Additionally, to gauge predictive value of models, we determined positive predictive value (PPV) and negative predictive value (NPV). PPV and NPV were estimated using formulas based on Bayes’ theorem as previously described [23] with 5% aneurysm prevalence, which is within the range of IA prevalence reported in the literature (3.2–7% [47–50]). The classification models were then independently evaluated in the testing cohort (n = 40), and classification results were compared to clinical diagnoses to calculate the true sensitivity, specificity, and accuracy for each model. ROC curves were constructed and AUCs, along with PPV and NPV, were used to assess the performance of each classifier. This was performed for algorithms trained on LASSO-selected features and the previous 26 features.

### Testing influence of clinical covariates on gene expression differences

While we randomly assigned samples to training or testing cohorts, this study was not cohort-controlled and used a large, heterogeneous population. Consequently, it is possible that factors other than IA status, such as demographics or comorbidities, could be affecting differential expression and model performance. To determine if patient characteristics influenced model performance, we first performed a chi-square test to determine if there were different rates in the aneurysm and control populations. We examined gender, hypertension, heart disease, stroke, high cholesterol, cancer, diabetes, arthritis, asthma, smoking status, and age. Additionally, we performed covariate matching in the MatchIt program in R to create 6 subclasses under default settings with similar distribution of covariates (age [60 and under vs over 60], sex, smoking status [non-smoker vs current smoker], hypertension, heart disease, stroke history, high cholesterol, cancer, diabetes, arthritis, asthma, and IA family history) for aneurysm and control populations [51, 52]. To create subclasses, we used a distance measure determined by a logistic regression model to estimate the propensity score. We then examined misclassification rate in each of the subclasses to determine if any group with a specific “covariate profile” was associated with greater misclassification.

### Bioinformatics

Gene ontology enrichment analysis was performed using Gene Ontology enRIchment anaLysis and visuaLizAtion tool (GORILLA) [53]. A background list of neutrophil expression from 3 healthy individuals (average fragments per kilobase million > 0.5) was used to compute hypergeometric statistics and assign significance to GO terms [7]. GO functions and processes with a p-value < 0.001 were reported. Ingenuity Pathway Analysis (IPA) software (Qiagen Inc., https://www.qiagenbioinformatics.com/products/ingenuity-pathway-analysis) was used to investigate networks associated with the differentially expressed genes identified by edgeR (q < 0.05, fold-change > 2) and those selected by LASSO during feature selection. Each gene identifier was mapped to its corresponding gene object in the Ingenuity Knowledge Base and overlaid onto a molecular network derived from information accumulated in the Knowledge Base. Gene networks were algorithmically generated based on their “connectivity” derived from known interactions between the products of these genes. Networks were considered significant if their p-scores were ≥ 15. Network score is calculated as p-score = −log10(p-value), so a score of 15 corresponds to a p-value of 1E-15 [54].

## Results

### Study population

We obtained and analyzed an additional 91 samples from individuals undergoing cerebral DSA that met data and RNA quality criteria. Combined with the 43 samples we previously analyzed, our total dataset was 134 neutrophil transcriptomes—55 from patients with IA and 79 from control patients. The characteristics of study population are presented in Table 1; detailed aneurysm characteristics in Additional file 3: Table S2. Overall, the 134 samples had an average 260/280 ratio of 2.04 (median: 2.05) and an average RIN of 6.7 (median: 6.7), as shown in the quality data reported in Additional file 4: Table S3. Patients with IA had 73 aneurysms (as 12 individuals had multiple IAs), which ranged in size from 1 to 19 mm measured by largest diameter on 2D images.

**Table 1:** Clinical characteristics of training and testing cohorts

	Training Cohort		Testing Cohort	
	Control (n = 55)	Aneurysm (n = 39)	Control (n = 24)	Aneurysm (n = 16)
Age (Mean ± SE)	62 ± 2.0	61 ± 1.7	59 ± 2.9	57 ± 3.3
Age [Median (Q1/Q3)]	66 (54/72)	60 (54/68)	59 (54/68)	58.5 (49.25/63.25)
Sex (% of patients)				
Female	56.36%	69.23%	50%	75%
Smoker (% of patients)				
Yes	10.91%	26.64%	20.83%	43.75%
Comorbidities (% of patients)				
Hypertension	61.82%	53.85%	54.17%	50%
Heart disease	30.91%	23.08%	25%	18.75%
High cholesterol	52.73%	48.72%	62.50%	50%
Stroke history	12.73%	10.26%	25%	0%
Diabetes	29.09%	17.95%	8.33%	31.25%
Arthritis	16.36%	30.77%	16.67%	18.75%

Clinical characteristics of the randomly-created training and testing cohorts. With the exception of age, these factors were quantified as binary data points. The clinical factors were retrieved from the patients’ medical records via the latest “Patient Medical History” form administered prior to imaging

### Differential RNA expression in neutrophils from patients with IA vs. controls

**Fig. 1:**
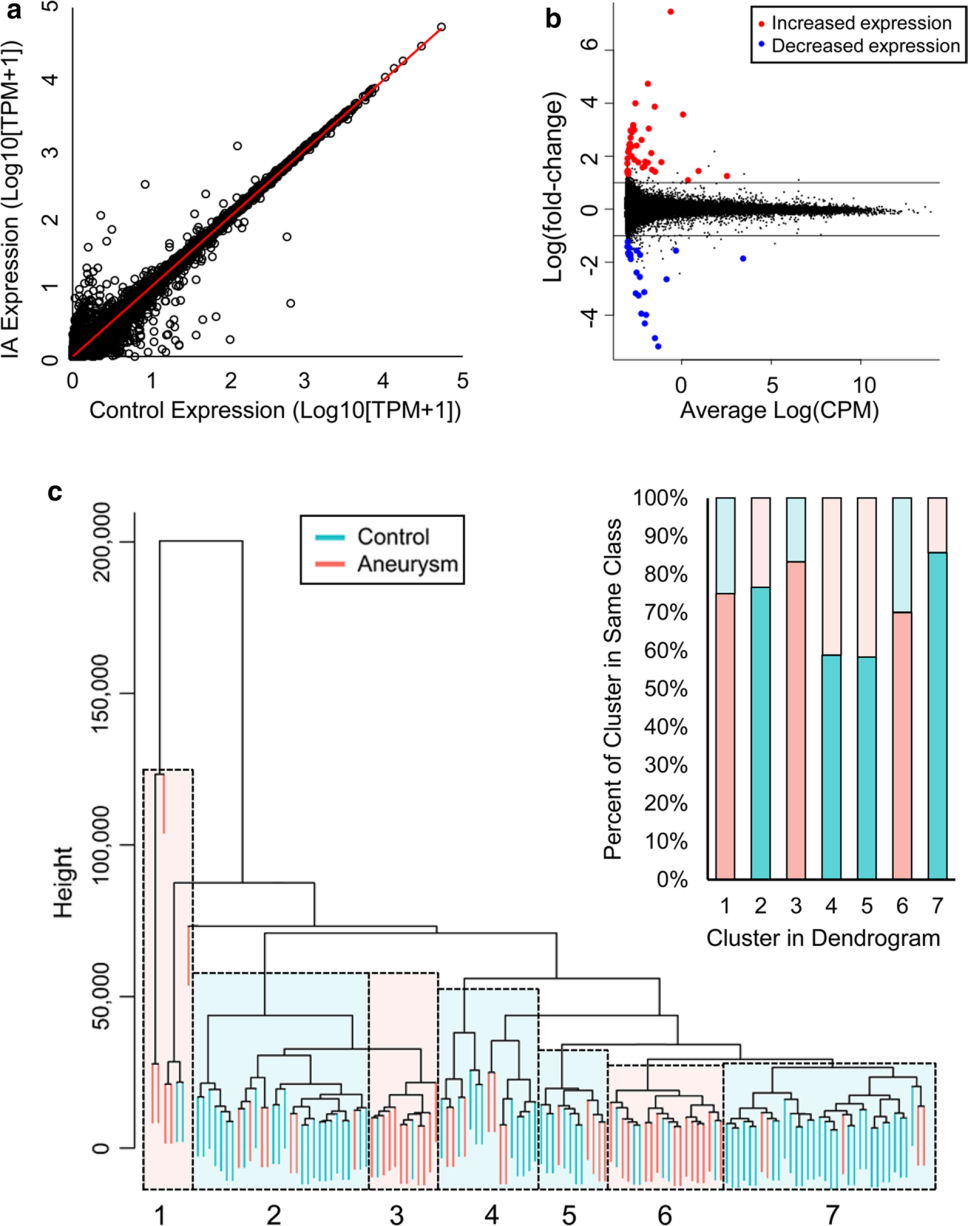
RNAseq data from whole dataset (n = 134). **a** The scatter plot demonstrates the dispersion in expression between the IA and control groups. **b** The volcano plot produced following edgeR analysis demonstrates that there are 65 differentially expressed genes. Red points are increased in IA group and blue points are decreased in IA group. **c** Clustering performed on all transcriptome data demonstrates several distinct clusters of IA and control samples. Overall, 73% of samples were assigned to the correct group

RNA sequencing data were used to identify differentially expressed neutrophil transcripts between IA and control groups. Overall, our sequencing experiments had an average of 55.06 million reads per sample and a 95% read mapping rate (or % aligned), as reported in Additional file 4: Table S3. The scatter plot in Fig. 1a shows neutrophil expression differences between IA patients and controls in terms of average fold-change in expression and significance level. Differential expression analysis in edgeR identified 65 transcripts that were significantly differentially expressed (q < 0.05, fold-change > 2) (red and blue points in Fig. 1b). Twenty-three genes showed lower expression in the IA group, and 42 showed higher expression. For all differentially expressed transcripts, correlation analysis demonstrated that RIN was not a significant co-variate; the maximum absolute Pearson correlation coefficient was 0.21, or a coefficient of determinization of R^2^ = 0.043 (range in R^2^_:_ 2.76E-6 to 0.043), and none had p-value < 0.01 (Additional file 5: Fig. S2A). Using all transcriptome data, we performed supervised hierarchical clustering to determine if gene expression in general could also discriminate patients with IAs from controls. On the dendrogram in Fig. 1c, samples from IA and control groups are separated. The dendrogram shows 7 clusters of primarily IA or control samples (highlighted sections). Overall, hierarchical clustering congregated 73% of the samples with their respective groups.

### Bioinformatics results

**Fig. 2:**
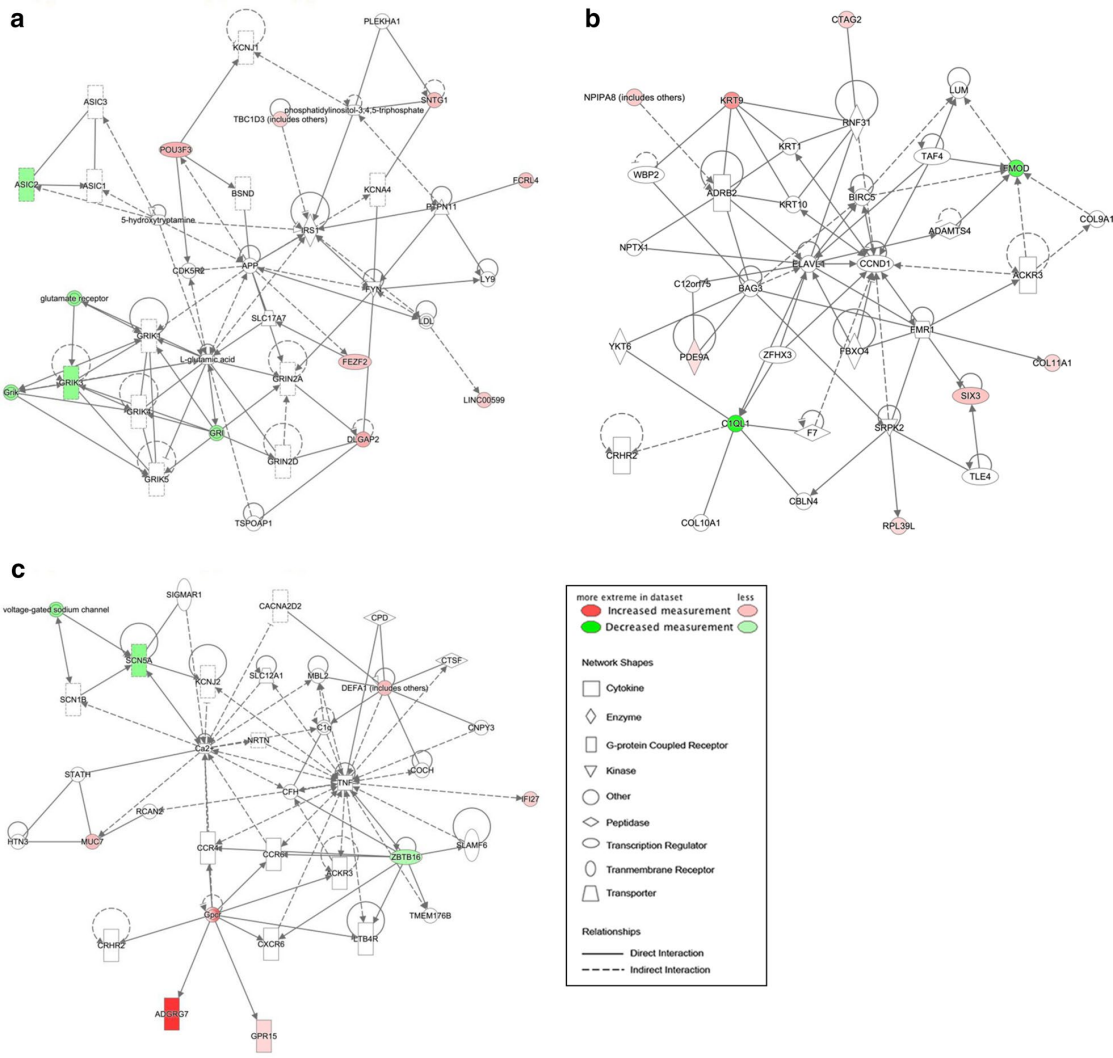
Networks derived from IPA of the 65 differentially expressed transcripts (q < 0.05, fold-change > 2). Transcripts with increased expression in IA are red; transcripts with lower expression in IA are green; fold-change is represented by intensity. **a** This network (p-score = 21) has related functions of cell-to-cell signaling and interaction, nervous system development and function, and cell morphology. **b** This network (p-score = 21) associated with dermatological diseases and conditions, organismal injury and abnormalities, and connective tissue development and function. **c** This network (p-score = 15) has ties to cell death and survival, connective tissue disorders, and inflammatory disease

To gain biological insight into the observed neutrophil RNA expression differences between IA and control groups, we performed bioinformatics analyses using gene set enrichment analysis and physiological pathway modeling. We used GORILLA to analyze the ontologies associated with the edgeR genes with increased and decreased expression in IA as compared to background of healthy individuals. Genes with increased expression in IA were associated with cell migration, cell motility, T cell migration, and lymphocyte migration processes. On the other hand, genes with decreased expression in IA had functions related to sodium channel activity, ion channel activity, and gated channel activity, as well as signaling and regulation of membrane potential processes. A full list of ontologies associated with edgeR genes is reported in Additional file 6: Table S4. IPA gene network analysis identified 3 significant networks with p-scores of 21, 21, and 15, respectively (Fig. 2). The first network was associated with cell morphology, cell-to-cell signaling and interaction, nervous system development and function, with hubs around *IRS1*, *GRIK1*, *GRIN2A*, and L-glutamic acid. The second network is associated with connective tissue development and function, dermatological diseases and conditions, organismal injury and abnormalities, with hubs of *ELAVL1*, *CCND1*, and *FMOD*. Finally, the last network was enriched for cell death and survival, connective tissue disorders, and inflammatory disease functions, reflected by a predominant hub being *TNF*. Associated molecules and diseases/functions for each network are listed in Additional file 7: Table S5.

### Verification of expression differences by RT-qPCR

**Fig. 3:**
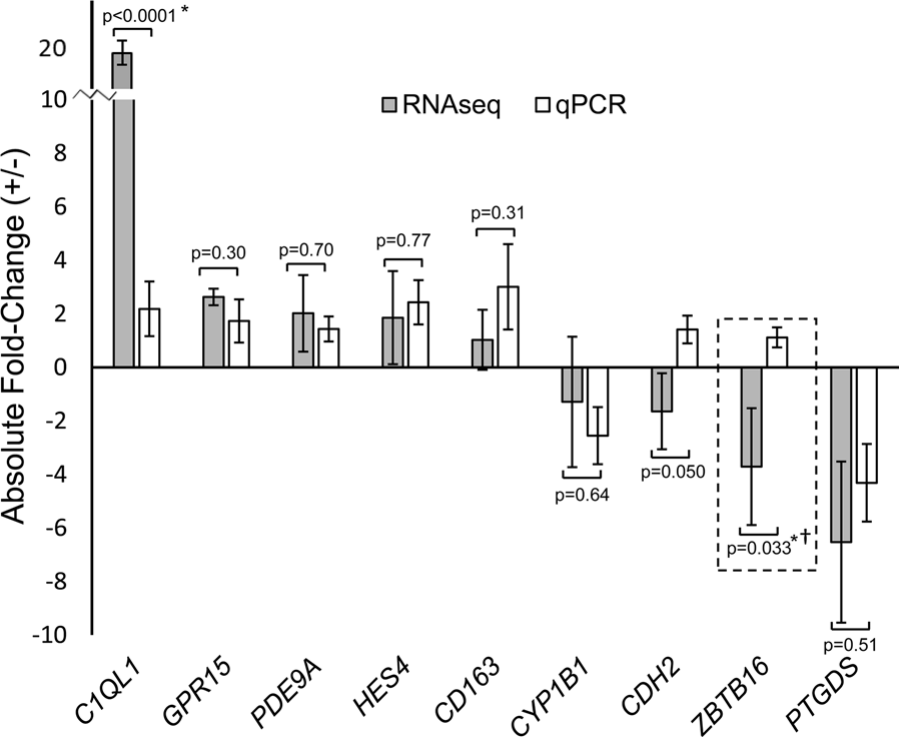
Verification of RNA-Sequencing data for 9 transcripts by qPCR. A total of 49 of the sequenced samples were analyzed by RT-qPCR, as the other samples did not have enough RNA for the additional reactions. Seven of the 9 transcripts in samples in a subset of patients had the same direction of expression difference on qPCR. There was a statistically significant difference in fold-change in expression (indicated by *) between RNAseq and qPCR for *C1QL1* and *ZBTB16*. Only *ZBTB16* had both a significant and different fold-change direction (indicated by †) than that calculated with RNA sequencing data. (Negative fold-change values calculated by negative inverse of fold-change, error bars = standard error.)

We confirmed expression differences of 9 prominent IA-associated transcripts (*C1QL1*, *GPR15*, *HES4*, *PVRL2*, *CD163, CYP1B1, CDH2, ZBTB16*, *PTGDS*) using RT-qPCR (qPCR was attempted on *PDE9A*, but results were not included due to low efficiency of the primer pairs; efficiency < 0.50). These genes were selected because they were prominently differentially expressed transcripts, i.e. were in the models we trained, were highly abundant in at least one cohort, or were significantly differentially expressed. This confirmation was performed in a subset of 49 patient samples, as one IA sample did not provide sufficient data for analysis across all genes and so that data is not included here. Figure 3 demonstrates that the expression differences between patients with and without IA were of the same direction and of similar magnitudes when calculated by both RNA sequencing and RT-qPCR, with the exception of *CDH2* and *ZBTB16*. There was a statistical difference between qPCR and RNA sequencing for *C1QL1* and *ZBTB16* (both p-values < 0.034). Only *ZBTB16* had both a significant and different direction in fold-change than that reported by RNA sequencing.

### Selected transcripts and model training

Feature selection using LASSO identified 37 IA-associated transcripts with significant expression in the training cohort, which were used to create models with 4 machine learning algorithms. Table 2 reports gene-specific accuracy, sensitivity, and specificity of the 37 model genes. For all transcripts selected by LASSO, correlation analysis also demonstrated that RIN was not a significant co-variate; the maximum absolute Pearson correlation coefficient was < 0.08 at 0.54 or R^2^ = 0.29 (range in R^2^_:_ 4.36E−6 to 0.29), although 11 genes had a p-value < 0.01 (Additional file 5: Fig. S2B).

**Table 2:** The 37 transcripts selected for classification model training

Gene	Gene ID	Accession #	Training Cohort			Testing Cohort		
			Acc	Sen	Spe	Acc	Sen	Spe
*AC011380.1*	–	AC011380	0.56	0.64	0.51	0.58	0.69	0.50
*C1QL1*^†^	10882	NM_006688.5	0.80	0.56	0.96	0.93	0.88	0.96
*CCDC42B*	387885	NM_001144872.2	0.59	0.05	0.96	0.65	0.19	0.96
*CEP295NL*^†^	100653515	NM_001243541.1	0.71	0.59	0.80	0.80	0.56	0.96
*CERS4*^†^	79603	NM_024552.3	0.79	0.72	0.84	0.85	0.88	0.83
*CLP1*	10978	NM_006831.3	0.57	0.00	0.98	0.58	0.00	0.96
*DCUN1D1*	54165	NM_020640.3	0.57	0.00	0.98	0.60	0.00	1.00
*EIF4EBP3*	8637	NM_003732.3	0.47	0.26	0.62	0.68	0.69	0.67
*FLT1*	2321	NM_002019.4	0.52	0.00	0.89	0.50	0.00	0.83
*GBGT1*^†^	26301	NM_021996.6	0.71	0.74	0.69	0.70	0.88	0.58
*GPR15*^†^	2838	NM_005290	0.79	0.79	0.78	0.93	1.00	0.88
*GPR157*	80045	NM_024980.5	0.57	0.00	0.98	0.60	0.00	1.00
*GTF2B*	2959	NM_001514.6	0.55	0.03	0.93	0.55	0.00	0.92
*HBB*	3043	NM_000518.5	0.79	1.00	0.64	0.58	0.94	0.33
*HIST1H4E*	8367	NM_003545.3	0.57	0.00	0.98	0.60	0.00	1.00
*HIST2H2AB*	317,72	NM_175065.2	0.57	0.00	0.98	0.60	0.00	1.00
*ISY1*	57461	NM_001199469.1	0.60	0.05	0.98	0.65	0.13	1.00
*KIAA1324*	57535	NM_020775.5	0.59	0.05	0.96	0.55	0.06	0.88
*KIAA1614*	57710	NM_020950.2	0.63	0.15	0.96	0.63	0.06	1.00
*LOC100129697*	100129697	NM_001290330.2	0.65	0.26	0.93	0.58	0.06	0.92
*LOC105377284*	105377284	XR_938891.2	0.59	0.05	0.96	0.65	0.13	1.00
*LRRN3*^†^	54674	NM_001099658.2	0.78	0.79	0.76	0.85	1.00	0.75
*MFSD6L*	162387	NM_152599.3	0.67	0.59	0.73	0.65	0.50	0.75
*MORC3*	23515	NM_015358.3	0.57	0.00	0.98	0.60	0.00	1.00
*MTRNR2L1*	100462977	NM_001190452.1	0.57	0.03	0.96	0.60	0.00	1.00
*NECAB1*	64168	NM_022351.5	0.62	0.15	0.95	0.58	0.06	0.92
*NEIL3*	55247	NM_018248.3	0.57	0.00	0.98	0.60	0.00	1.00
*PDCD10*	11235	NM_007217.4	0.57	0.00	0.98	0.60	0.00	1.00
*PGM5*	5239	NM_021965.4	0.55	0.00	0.95	0.50	0.00	0.83
*RFFL*	117584	NR_037713.1	0.57	0.00	0.98	0.58	0.00	0.96
*SDCBP2*	27111	NM_080489.5	0.71	0.33	0.98	0.65	0.31	0.88
*SMIM8*	57150	NM_001042493.3	0.57	0.03	0.96	0.50	0.00	0.83
*SYP*	6855	NM_003179.2	0.59	0.05	0.96	0.63	0.13	0.96
*TGS1*^†^	96764	NM_024831.7	0.80	0.79	0.80	0.78	0.94	0.67
*TMC4*	147798	NM_001145303.2	0.64	0.49	0.75	0.63	0.44	0.75
*USF1*	7391	NM_007122.5	0.59	0.03	0.98	0.60	0.06	0.96
*UTY*	7404	NM_182660.1	0.57	0.00	0.98	0.60	0.00	1.00

*Acc* accuracy, *Sen* sensitivity, *Spe* specificity

We show the per-transcript performance in the training and testing dataset. Transcripts with high accuracy (> 0.70) in both training and testing cohorts are denoted by ^†^

**Fig. 4:**
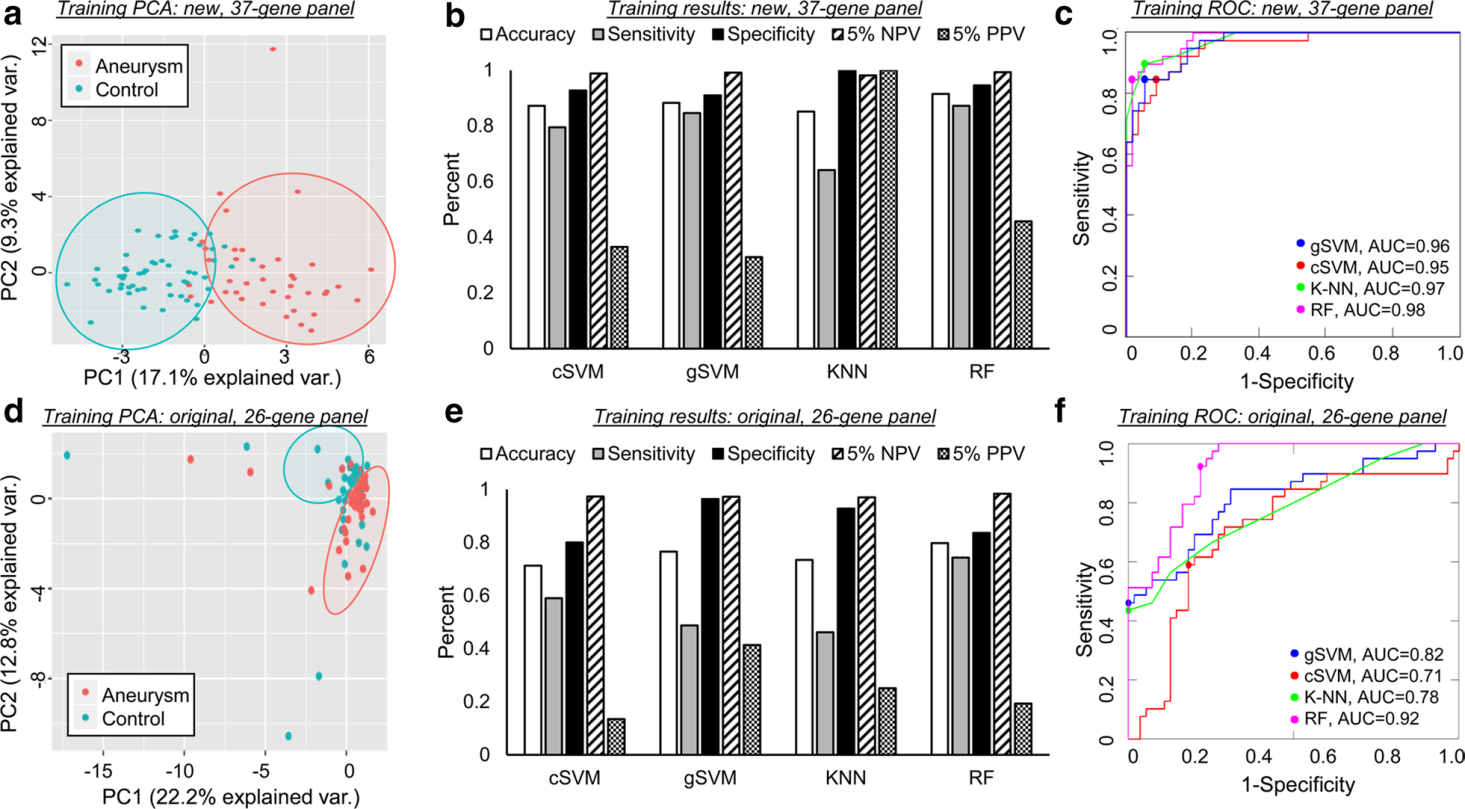
Models’ performance in the training dataset. **a** PCA using the 37 selected transcripts demonstrated clear separation between samples from patients with IA and those from controls. **b** Estimation of model performance during LOO C-V in the training cohort demonstrated that models performed with an accuracy of 0.85–0.91. Considering a 5% prevalence of IA, PPV ranged from 0.33–1 and NPV ranged from 0.98 to 0.99. **c** ROC analysis showed that all models had AUCs ≥ 0.95. **d** PCA using the 26 previously-identified transcripts demonstrated inferior separation between IA and control cases. **e** Estimation of model performance during LOO C-V in the training cohort demonstrated that models performed with an accuracy of 0.71–0.80. Considering a 5% prevalence, PPV and NPV ranged from 0.13–0.41 and 0.97–0.98, respectively. **f** ROC analysis also showed subpar performance compared to newly identified transcripts (AUC range 0.71–0.92). (AUC = area under the ROC curve, C-V = cross validation, cSVM = cubic support vector machines, gSVM = Gaussian support vector machines, KNN = k-nearest neighbors, LOO = leave-one-out, NPV = negative predictive value, PCA = principal component analysis, PPV = positive predictive value, RF = random forests, ROC = receiver operator characteristic)

Figure 4 demonstrates PCA (a and d), performance metrics (b and e), and ROC curves (c and f) for the training of four types of machine learning models that utilize either the new 37-transcript panel or our original 26-gene panel. The PCA in Fig. 4a illustrates these transcripts’ ability to clearly separate aneurysm samples from control in training cohort. Compared to the PCA using the 26 previously identified genes in Fig. 4d, it is visually evident that the transcripts identified by LASSO were able to better separate IA and control groups.

Sensitivity, specificity, accuracy, NPV, and PPV estimated by LOO cross-validation in the training cohort are reported in Fig. 4b for models using the new 37-transcript panel. Each classification method achieved high performance, with accuracies that ranged from 0.85 to 0.91. Evaluation by ROC curve analysis showed a range in AUCs from 0.95 to 0.98 (Fig. 4c) across all methods. All models had high NPV of approximately 1 (0.98–1). Random Forest outperformed K-Nearest Neighbor and both Support Vector Machine algorithms, with a sensitivity of 0.87, specificity of 0.95, accuracy of 0.92, AUC of 0.98, NPV of 0.99, and PPV of 0.46. Figure 4e reports performances of the 4 classification models trained with the 26 previously-identified genes. Sensitivity, specificity, accuracy, NPV, and PPV were estimated by LOO cross-validation in the training cohort. AUCs ranged from 0.71 to 0.92, as shown in Fig. 4f. Overall performance in the training cohort was superior using the transcripts selected by LASSO; all metrics (accuracy, sensitivity, specificity, AUC, 5% PPV, 5% NPV) when averaged across the 4 models were greater using the new 37-gene panel.

### Predictive models of IA have high performance and high NPV in testing

**Fig. 5:**
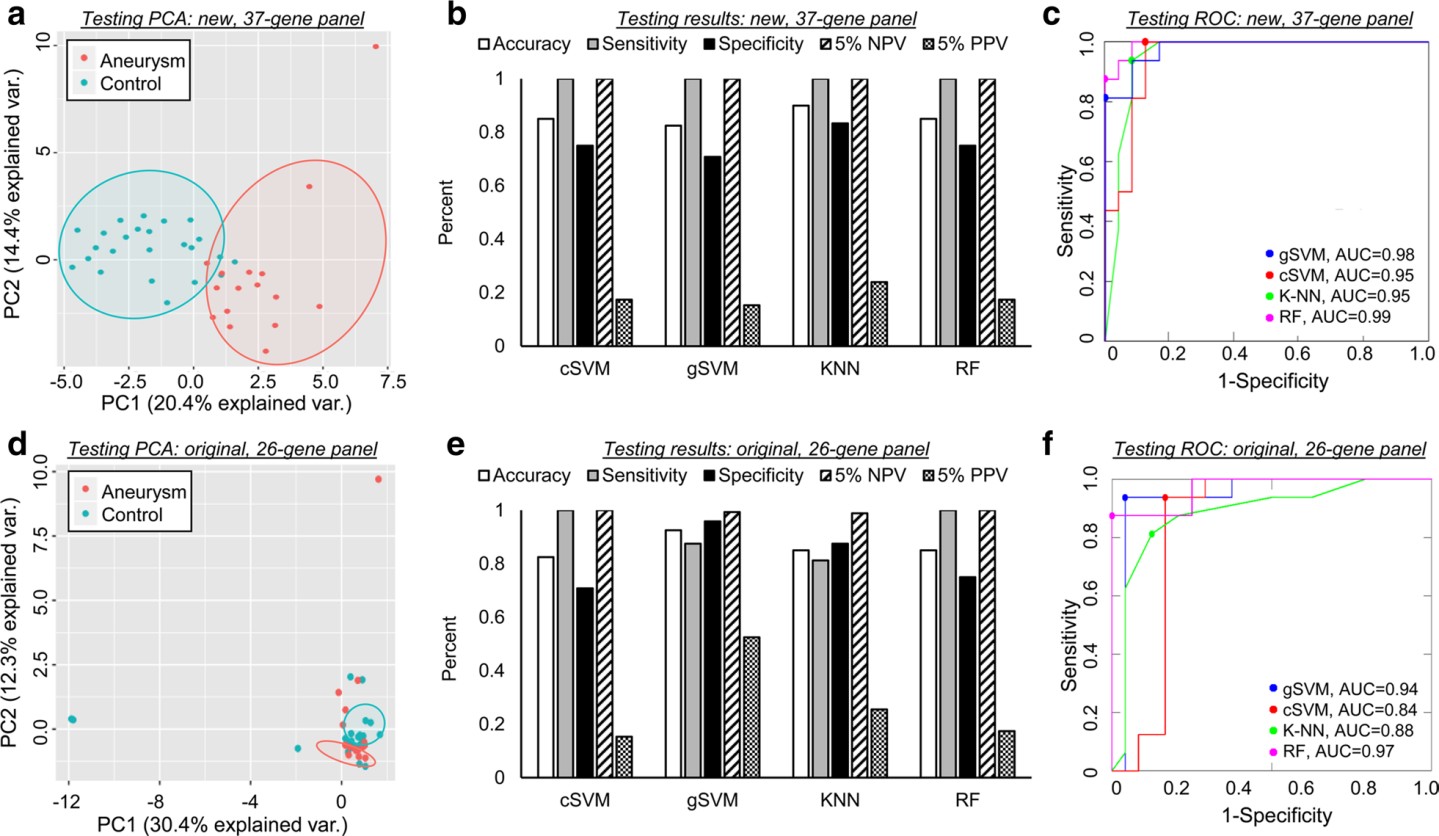
Models’ performance in the testing dataset. **a** PCA using the 37 selected transcripts in this independent dataset also demonstrated strong separation between samples from patients with IA and from controls. **b** Assessment of true model performance showed that models performed with an accuracy of 0.83–0.90. In this dataset all models had a sensitivity of 1. At 5% IA prevalence, the PPV ranged from 0.15 to 0.24 and NPV was 1 for all models. **c** ROC analysis showed that all models again had AUCs ≥ 0.95. **d** PCA using the 26 previously-identified transcripts demonstrated mediocre separation between IA and control cases. **e** Estimation of model performance in the testing cohort demonstrated that models performed with an accuracy of 0.83–0.93. Considering a 5% prevalence, PPV and NPV ranged from 0.15–0.52 and 0.99–1, respectively. **f** ROC analysis also showed inferior performance compared to newly identified transcripts (AUC range 0.84–0.97). (AUC = area under the ROC curve, C-V = cross validation, cSVM = cubic support vector machines, gSVM = Gaussian support vector machines, KNN = k-nearest neighbors, LOO = leave-one-out, NPV = negative predictive value, PCA = principal component analysis, PPV = positive predictive value, RF = random forests, ROC = receiver operator characteristic)

Figure 5 demonstrates PCA (a and d), performance metrics (b and e), and ROC curves (c and f) for the testing of the machine learning models, which utilize either the new 37-transcript panel or our original 26-gene panel. PCA performed on the testing data (Fig. 5a) shows that the 37 transcripts could discriminate patients with IAs from controls. The separation between classes was more obvious using the 37 newly-identified transcripts than the 26 previously-identified transcripts (Fig. 5d). Using the 37 features selected by LASSO, the models predicted aneurysm status in the testing cohort with accuracies ranging from 0.83 to 0.90 (Fig. 5b). The ROC analysis in Fig. 5c shows that model AUCs ranged from 0.95 to 0.99. In the testing cohort, the Random Forest model again performed well, with a sensitivity of 1.0, specificity of 0.75, accuracy of 0.85, and AUC of 0.99. The performance of the previously identified 26-gene panel (Fig. 5e, f) was similar to that of the 37-gene panel in the testing cohort with accuracies ranging from 0.83 to 0.93 and AUCs of 0.84–0.97. While average accuracy for the 4 models was the same (86%) using the 37-gene panel identified by LASSO and the 26-gene panel previously identified, the models using the 37 LASSO features had greater average AUC (0.97 vs 0.91).

### Bioinformatics of LASSO-selected transcripts

**Fig. 6:**
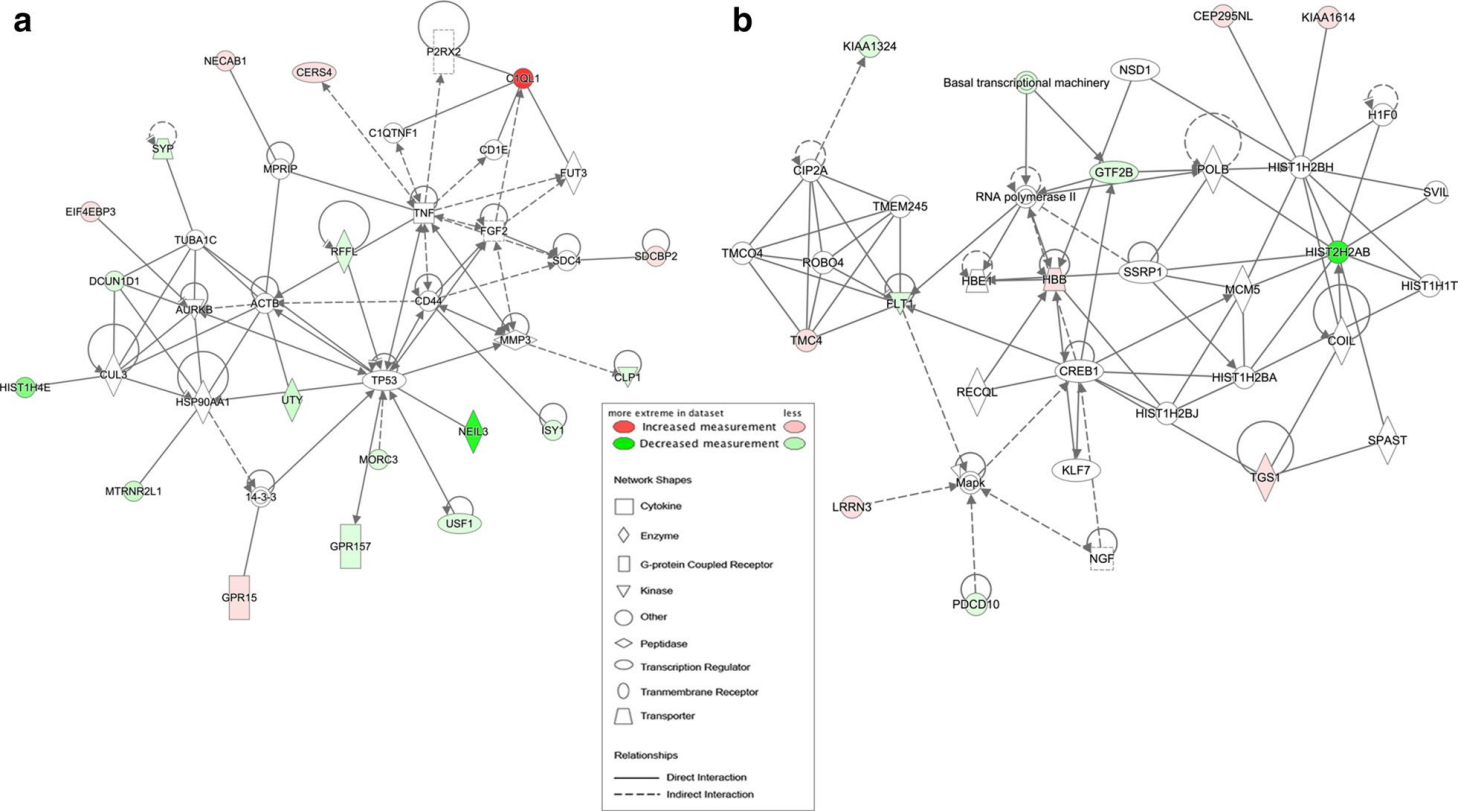
Networks derived from IPA of the 37 genes identified by LASSO. Transcripts with increased expression in IA are red; transcripts with lower expression in IA are green; fold-change is represented by intensity. **a** This network (p-score = 47) affiliated with cancer, cellular movement, and connective tissue disorders. **b** This network (p-score = 25) has associated functions of cell cycle, cellular assembly and organization, DNA replication, recombination, and repair

To investigate how the biological underpinnings of IA specifically influence the genes selected by LASSO, we performed bioinformatics analyses using only the 37 new panel genes (n = 23 with decreased expression in IA group, n = 14 with increased expression in IA group). Model genes with decreased expression in the IA group were associated with negative regulation of execution phase of apoptosis, negative regulation of endothelial cell proliferation, and regulation of execution phase of apoptosis (Additional file 8: Table S6). However, model genes with increased expression in the IA group did not return any significant functions or processes. Two networks using all 37 genes produced by IPA had significant p-scores (equal to 47 and 25). The first network showed hubs around *TNF* and *MMP3* and was associated with cancer, cellular movement, and connective tissue disorders. The second had hubs around *HBB* and *MAPK* and was associated with cell cycle, cellular assembly and organization, DNA replication, recombination, and repair. We note *TNF* was incorporated in networks generated using both edgeR and LASSO gene sets. See Fig. 6 and Additional file 9: Table S7 for details on these networks, including associated molecules and top diseases and functions.

### Presence of clinical covariates and effect on model performance

Table 3 shows the rates of demographics and comorbidities in aneurysm and control populations. Only smoking was significantly higher in the IA population (p = 0.017), which can be expected as it is a well-known risk factor for IA and IA rupture [55]. We created 5 subclasses using MatchIt as there were too few samples in the 6th subclass. Misclassification by the 37-gene prediction model for each subclass ranged from 8 to 19%, indicating no one subclass could be driving misclassification.

**Table 3:** Clinical characteristic differences in entire population

	Control (n = 79)	Aneurysm (n = 55)	Chi-square test
Age (Mean ± SE)	61 ± 1.7	60 ± 1.5	(age 60 cutoff) 0.243
Age [Median (Q1/Q3)]	65 (54/72)	60 (54/67)	
Sex (% of patients)			
Female	54.43%	70.91%	0.054
Smoking (% of patients)			
Current	13.92%	30.91%	0.017^†^
Comorbidities (% of patients)			
Arthritis	16.46%	27.27%	0.130
Asthma	7.59%	18.18%	0.063
Cancer	11.39%	9.09%	0.668
Diabetes	22.78%	21.82%	0.895
Heart disease	29.11%	21.82%	0.344
High cholesterol	55.70%	49.09%	0.451
Hypertension	59.49%	52.73%	0.437
IA family history	7.69%	12.73%	0.336
Stroke history	18.99%	9.09%	0.114

None of the reported covariates were significantly different in either group (chi-square test < 0.05) except for smoking^†^

## Discussion

### More robust machine learning strategy improves biomarker performance

In this study, we implemented a new machine learning strategy for IA biomarker discovery, which consisted of a larger dataset (94 training, 40 testing), LASSO for feature selection, and more robust algorithms, K-Nearest Neighbor, Random Forest, and Support Vector Machine with cubic and Gaussian kernels. Our larger dataset and LASSO feature selection led to a new panel of 37 genes to use in IA predictive models. Two genes of these 37 genes, *C1QL1* and *TGS1*, were also in our previously-discovered 26-gene panel. The new learning algorithms trained using the 37 genes all performed very well in the testing cohort with accuracies of 0.83–0.90 and AUCs of 0.95–0.99, a marked increase over our previous algorithms. Interestingly, all 4 new models had an NPV of 1, indicating that in the testing dataset there were no false negatives. This may be important for future applications of these biomarkers as a prescreen, since false negatives would be particularly deleterious.

To examine how the increased sample size and improved algorithms affected model performance, we retrained the previous 26-gene panel using the new algorithms in the current, larger dataset. The performance of the retrained models in the testing set (n = 40) using the 26-gene panel improved from our previous study with accuracies ranging from 0.83 to 0.93 and AUCs of 0.84–0.97. Despite this increase in performance using the new algorithms, models using the previously identified 26 genes still fell short of those using the newly identified 37 genes; the average testing AUC using 26 genes was 0.91 compared to 0.97 when using 37 genes. This suggests that the 37 features identified by LASSO are more reliable for IA prediction than the 26 selected by filtering in our last study.

Improved IA prediction could be attributed to our increased sample size, which afforded several advantages. First, it allowed us to use LASSO to identify features instead of simple filtering methods. Thresholding filters, as we had previously used, consider each gene independently, which can neglect groups of genes that function together in pathophysiologic mechanisms and could be useful as a biomarker. Filtering methods can also select highly correlated, redundant genes that increase the number of features required to make accurate predictions. HSIC LASSO, a nonlinear feature selection method, overcomes these issues and identifies combinations of non-redundant genes with strong dependence on disease status. Implementing LASSO in the training dataset identified 37 unique IA-associated genes, two of which (*C1QL1*, *TGS1*) had also been identified as part of the 26-gene panel in our last expression profiling study [23]. The identification of non-redundant features may be one reason why the biomarkers created in this study outperform our past efforts, as some of the 26 features (with the exception of *C1QL1* and *TGS1*) may have ultimately been uninformative for classification.

Secondly, a larger sample size also enabled us to leverage more complex machine learning models, namely Support Vector Machine and Random Forests which perform better in larger datasets [56]. In our previous effort we did implement Support Vector Machine, but only achieved a testing accuracy of 0.70, possibly because the training dataset contained only 30 patients [23]. In this larger study we were able to achieve an accuracy of 0.85 for Support Vector Machine (Gaussian kernel). Nevertheless, we found that in our data Random Forest consistently performed the best, with a testing accuracy of 0.85 and AUC of 0.99. Both Random Forests and K-Nearest Neighbors are weighted neighbors schemes. However, the K-Nearest Neighbors algorithm may have had poorer performance because this classifier simply uses the training data for prediction instead of learning a discriminative rule. The performance of the K-Nearest Neighbors classifier is reliant on the quality of the training data, which in the case of transcriptomes derived from human samples may be noisy. However, this problem is well-solved in Random Forest. Through the random sampling process, Random Forest handles outliers by binning them. Also, by averaging the decision trees, the Random Forest method provides a low bias and moderate variance model, which improves the generalizability of the output model. In other words, Random Forest not only attains a good performance in the training data but also performs well in unknown (testing) data. And while Support Vector Machine performed well here, Random Forest likely surpassed Support Vector Machine by avoiding overfitting and achieving better predictive power.

We note that increasing sample size may have introduced more variability in our data due to a larger, heterogeneous population that was not cohort-controlled. For example, in our entire population we found smoking was significantly higher in patients with IA (χ = 0.017), which may be because smoking is a well-known risk factor for IA formation and rupture [57–59]. Indeed two genes in our model, *LRRN3* and *GPR15*, are among the top differentially expressed genes in blood between current and never smokers according to a meta-analysis by Huan et al. [60] Their presence in our predictive model may be because of the higher proportion of smokers in IA group or because these genes are capturing biological mechanisms related to smoking that are important in IA pathogenesis, such as endothelial dysfunction [61–63]. Still, when we performed covariate analysis using MatchIt to create subgroups with similar distributions of covariates between IA and control groups, we found that no one subgroup had significantly higher misclassification rates. For instance, 61% of all subjects in “Subclass 5” were smokers, and this subgroup had a misclassification rate of 13%. Yet, “Subclass 1”, which had 0% smokers, had a misclassification rate of 14%. These results suggest that our prediction models may not be affected greatly by covariate imbalance, albeit testing this in even larger cohorts will be needed to confirm these results.

### Complex role of circulating neutrophils in intracranial aneurysm

Inflammation is widely-recognized to play a central role in the pathophysiology of IA [64–66]. It is commonly thought that in IA neutrophils are recruited to the sac, where they infiltrate the wall and coordinate the inflammatory responses [64, 67, 68]. In this study, gene ontology enrichment analysis showed that genes with higher expression in IA identified by edgeR in the entire dataset were related to cell migration and lymphocyte migration ontologies. These processes, which were also observed in neutrophils from patients with IAs in our previous studies [22, 23], increase upon peripheral activation and prompt inflammatory cell migration and infiltration of diseased tissue [64, 69]. IPA analysis mirrored these results, showing 3 significant networks, 2 of which were involved in activation-related processes: cell-to-cell signaling and interaction, and inflammatory disease function. Interestingly, one of the largest nodes of gene connectivity in all the networks was *TNF*, a proinflammatory cytokine with many functions including regulation of cell proliferation and apoptosis. *TNF* has been shown to have a mechanistic role in IA formation in animal models, [70] and an increased presence in human IA tissue compared to superficial temporal artery control tissue [71]. In this network, *TNF* has a predicted connection to *DEFA1*, which was significantly elevated in neutrophils of IA patients. Higher levels of this cytotoxic defensin protein that is contained within neutrophil granules have been reported in IA tissue, suggesting that production of this protein may occur peripherally before neutrophils enter the IA wall [72]. Here the molecules *CCR4* (which is a receptor of *MIP-1*, *RANTES*, *CCL17*, and *MCP-1*) [73, 74] and *CCR6* (which is a receptor of *MIP-3α*) [75] were also related to the *TNF* node. We suspect that these receptors, which play a role in dendritic and T cell migration and recruitment during inflammation, [76] may coordinate inflammatory cell migration once expressed in aneurysm tissue.

We also observed the dysregulation of inflammation and a potential role of *TNF* in our bioinformatics analyses of model genes selected by LASSO in the training dataset. *TNF* was a hub of connectivity in networks created using the LASSO genes. In these networks, we observed an indirect relationship between *TNF* and the complement system (i.e. *C1QTNF1*), which is also associated with *C1QL1* (one of 37 model genes). This may be because complement activation plays a critical role in the inflammatory response, [77] has been implicated in IA wall degradation and rupture, [78] and involves proteins that are increased in human IA tissue (including *CFB*, *CFH*, *C1Q*, and *C3AR1* [79]). We suspect that the complement alternative pathway may be one mechanism through which neutrophils become activated as it can amplify activation through a positive feedback mechanism [80]. In addition to complement members, the *TNF* node was also related to *CD44*, a cell surface glycoprotein critical to neutrophil recruitment during inflammation. Because neutrophils interact with *CD44*, *PSGL-1*, and *E-selectin ligand 1* as they roll along activated endothelial cells, this result may reflect neutrophils transmigrating into inflamed endothelium [81]. Our data shows *TNF* may also interact with the transcription factor *TP53*, a node with connections to numerous molecules, many of which have decreased expression. *TP53* plays a variety of roles in inflammation, such as acting on the NF- κB pathway [82]. NF- κB is a key transcription factor in the pathogenesis of IA, as it controls the inflammatory responses in the vessel wall [83]. The activation of NF-κB leads to the upregulation of *MCP-1* and *VCAM* that function to recruit monocytes to the IA lumen, where they become macrophages and secrete MMP-2 and MMP-9 to degrade the extracellular matrix [84]. Overall, our bioinformatics analyses of genes selected by LASSO, while not overlapping greatly with the differentially expressed genes selected by edgeR in the entire dataset (with the exception of *C1QL1*, *GRP15*), show that the biology of neutrophil activation and inflammation responses are captured by the IA prediction model gene panel.

In addition to neutrophil activation and heightened inflammatory signaling, we observed other aberrant neutrophil functions not specifically characterized in IA, including our previous studies [22, 23]. In genes identified in the whole dataset by edgeR, gene ontology enrichment analysis showed that the differentially expressed genes with decreased expression in IA had functions related to sodium channel activity, ion channel activity, and gated channel activity, as well as signaling and regulation of membrane potential processes (*ASIC2*, *GRIK3*, *SCN5A*). *GRIK3*, glutamate receptor 7, is particularly interesting as glutamate is a chemotactic factor for neutrophils after injury or infection [85]. Glutamate binding to its receptors can trigger release of cytokines and MMPs and can activate immune responses, all critical processes in IA [86, 87]. Future studies are needed to better understand how these channel activities impact IA pathogenesis.

New ontologies were also captured using the genes identified by LASSO in the training dataset. Using the LASSO genes with lower expression in IA, we found dysregulation of apoptosis as gene ontology enrichment analysis reported both negative regulation of execution phase of apoptosis and regulation of execution phase of apoptosis. These were associated with *RFFL*, which has been shown to be related to *TNF* signaling [88]. Ontologies were also related to *MTRNR2L1 (MTRNR2-Like 1)* that may function similarly to Humanin (*MTRNR2*), which is protective against cell death by suppressing several apoptotic pathways. Several studies have shown that, in this way, Humanin may be a neuroprotective factor that can influence Alzheimer’s disease and other angiopathy-associated neurodegenerative diseases [89, 90]. Perhaps in our study, dysregulated *MTRNR2L1* expression (as well as *TP53*, which also induces apoptosis) [91, 92] may be responsible for increasing the lifespan of neutrophils, which would provide further evidence of neutrophil activation in IA. These results are echoed in the blood profiling study of IA published by Jin et al. [93]. They reported hsa-miR-21, an upregulated miRNA in IA serum, induces apoptosis by extracellular signals, potentially triggering more apoptotic reactions to facilitate the medial thinning and destructive remodeling, a hallmark of IA pathogenesis [94–97]. Overall, we suspect that capturing neutrophil activation and inflammation responses involved in IA is the reason why the 37-gene panel was able to detect IA.

### Limitations

In this study, we increased sample size from our previous study by adding 94 samples to 40 samples we previously analyzed. However, these two batches used different versions of the Illumina kit for library preparation, which necessitated the implementation of batch effect correction that could potentially have introduced bias or skewed our dataset [98]. Secondly, RNA sequencing was performed on samples with a wide range of RIN values. While we demonstrated that RIN was not a significant co-variant of differential expression, in the future methods such as DegNorm developed by Xiong et al. [30] could be implemented to correct for expression variation due to differences in RNA degradation and potentially yield more accurate results. Thirdly, all samples were recruited from patients receiving cerebral imaging at a single center, which may introduce selection bias. Future studies are needed to validate our predictive models using broader patient populations from multiple centers. Fourthly, inflammatory or vascular diseases other than IA could affect model prediction. Larger studies with multiple control groups of individuals with other vascular and inflammatory conditions are needed to refine our model. Lastly, more rigorous qPCR performed specifically on model genes with more efficient probes (primers) will be needed to translate this gene panel into an assay that can show linearity in output and reproducibility over technical replicates.

## Conclusions

We improved IA predictive model performance from circulating neutrophil transcripts by using LASSO for feature selection and powerful machine learning techniques in a large dataset. The Random Forest algorithm performed the best with a testing AUC of 0.99. Bioinformatics using all 134 samples implicated inflammation through *TNF* and neutrophil activation as key processes in IA. IPA networks using the 37 LASSO-selected genes also reflected these increased inflammatory and signaling pathways. Comorbidities and demographics did not significantly affect IA prediction. Future studies are needed to validate these predictive models.

## Supplementary information

**Additional file 1: Table S1.** Primers used for qPCR.

**Additional file 2: Figure S1.** Stability of housekeeping genes in RNA sequencing and qPCR.

**Additional file 3: Table S2.** Characteristics of 73 aneurysms in all patients with intracranial aneurysms.

**Additional file 4: Table S3.** Batch Assignment, RNA QC, and sequencing QC for the testing and training cohorts.

**Additional file 5: Figure S2.** Co-variate correlation analysis between RNA quality (RIN) and differentially expressed genes.

**Additional file 6: Table S4.** GORILLA enriched ontologies for edgeR genes.

**Additional file 7: Table S5.** Transcripts and functions for the significant networks constructed by Ingenuity Pathway Analysis (IPA) using genes identified by edgeR.

**Additional file 8: Table S6.** GORILLA enriched ontologies for LASSO genes.

**Additional file 9: Table S7.** Transcripts and functions for the significant networks constructed by Ingenuity Pathway Analysis (IPA) using genes identified by LASSO.

## Data Availability

The datasets used in the current study are available from the corresponding author on reasonable request.

## Abbreviations

AUC: Area under ROC curve
BAM: Binary alignment map
BCL: Per-cycle base-call
CTA: Computed tomography angiography
DSA: Digital subtraction angiography
ECM: Extracellular matrix
FDR: False discovery rate
GO: Gene ontology
GORILLA: Gene Ontology enRIchment anaLysis and visuaLizAtion
HSIC: Hilbert–Schmidt independence criterion
IA: Intracranial aneurysm
IPA: Ingenuity pathway analysis
LASSO: Least absolute shrinkage and selection operator
LOO: Leave one-out
MPO: Myeloperoxidase
MRA: Magnetic resonance angiography
MRI: Magnetic resonance imaging
NGAL: Neutrophil gelatinase-associated lipocalin
NPV: Negative predictive value
PCA: Principal component analysis
PPV: Positive predictive value
qPCR: Quantitative polymerase chain reaction
RIN: RNA integrity
ROC: Receiver-operating-characteristic
ROS: Reactive oxygen species
RT-qPCR: Real-time quantitative polymerase chain reaction
TPM: Transcript per million
