# Supplementary material for: Classification models using circulating neutrophil transcripts can detect unruptured intracranial aneurysm
Source: J Transl Med. 2020 Oct 15;18:392. doi: 10.1186/s12967-020-02550-2 (PMC7565814; doi:10.1186/s12967-020-02550-2)
Supplement: Supplementary file 1 — Additional file 1: Table S1. Primers used for qPCR. [file 12967_2020_2550_MOESM1_ESM.docx]

**Supplemental Table 1. Primers used for qPCR.***

| **Transcript** | **Primer Sequence** | **Annealing**  **Temp. (^o^C)** | **Efficiency** | **PCR Prod.**  **Length (bp)** |
| --- | --- | --- | --- | --- |
| *C1QL1* | 5'-GCAACATTCCCGGCACCTAC-3' | 60.0 | 1.0 | 151 |
|  | 5'-TTGCTGGCGTAGTCGTAGTTC-3' |  |  |  |
| *GPR15* | 5'-TTTGGTGATGGACCCAGAAG-3' | 60.0 | 1.0 | 155 |
|  | 5'-CCAGCACTCCAGTCAGGAAC-3' |  |  |  |
| *PDE9A* | 5'-GCAGATCCGACAGGGAATG-3' | 60.0 | >1.0 | 131 |
|  | 5'-TCAGCAGGGTCATGTGCTC-3' |  |  |  |
| *HES4* | 5'-GGTGACGGCCGCGCTCAG-3' | 60.0 | 1.0 | 103 |
|  | 5'-CTCGCAGCCGGCCAGGAAG-3' |  |  |  |
| *CD163* | 5'-ATTACCTGCTCAGCCCACAG-3' | 60.0 | 0.84 | 130 |
|  | 5'-TGGCAGCTTCCAGAGAGAAG-3' |  |  |  |
| *CYP1B1* | 5'-CTGCTCCTCCTCTTCACCAG-3' | 60.0 | 0.95 | 132 |
|  | 5'-AAGGAAGGCCAGGACATAGG-3' |  |  |  |
| *CDH2* | 5'-CAGGGGACCTTTTCCTCAAG-3' | 60.0 | 0.84 | 199 |
|  | 5'-ATGTGCCCTCAAATGAAACC-3' |  |  |  |
| *ZBTB16* | 5'-TGTGGAGCAGCACAGGAAG-3' | 60.0 | 1.0 | 206 |
|  | 5'-CAGTGCCAGTATGGGTCTGC-3' |  |  |  |
| *PTGDS* | 5'- CACCCTCTACAGCCGAACC-3' | 60.0 | 1.0 | 131 |
|  | 5'-GTTCCGTCATGCACTTATCG-3' |  |  |  |
| *HPRT1* | 5'-TGGCGTCGTGATTAGTGATG-3' | 60.0 | 0.82 | 131 |
| Housekeeping | 5'-CAAGACGTTCAGTCCTGTCC-3' |  |  |  |
| GAPDH | 5'-CGCTCTCTGCTCCTCCTGTT-3' | 60.0 | 1.1 | 81 |
| Housekeeping | 5'-CCATGGTGTCTGAGCGATGT-3' |  |  |  |
| GPI | 5'-AGGCTGCTGCCACATAAGGT-3' | 60.0 | 0.95 | 240 |
| Housekeeping | 5'-AGCGTCGTGAGAGGTCACTTG-3' |  |  |  |

*Primers were selected using Primer3 and NCBI’s Primer Blast. (bp=base pair, Prod.=product, qPCR=quantitative polymerase chain reaction, Temp.=temperature)
