# Supplementary material for: Classification models using circulating neutrophil transcripts can detect unruptured intracranial aneurysm
Source: J Transl Med. 2020 Oct 15;18:392. doi: 10.1186/s12967-020-02550-2 (PMC7565814; doi:10.1186/s12967-020-02550-2)
Supplement: Supplementary file 2 — Additional file 2: Figure S1. Stability of housekeeping genes in RNA sequencing and qPCR. [file 12967_2020_2550_MOESM2_ESM.docx]

**
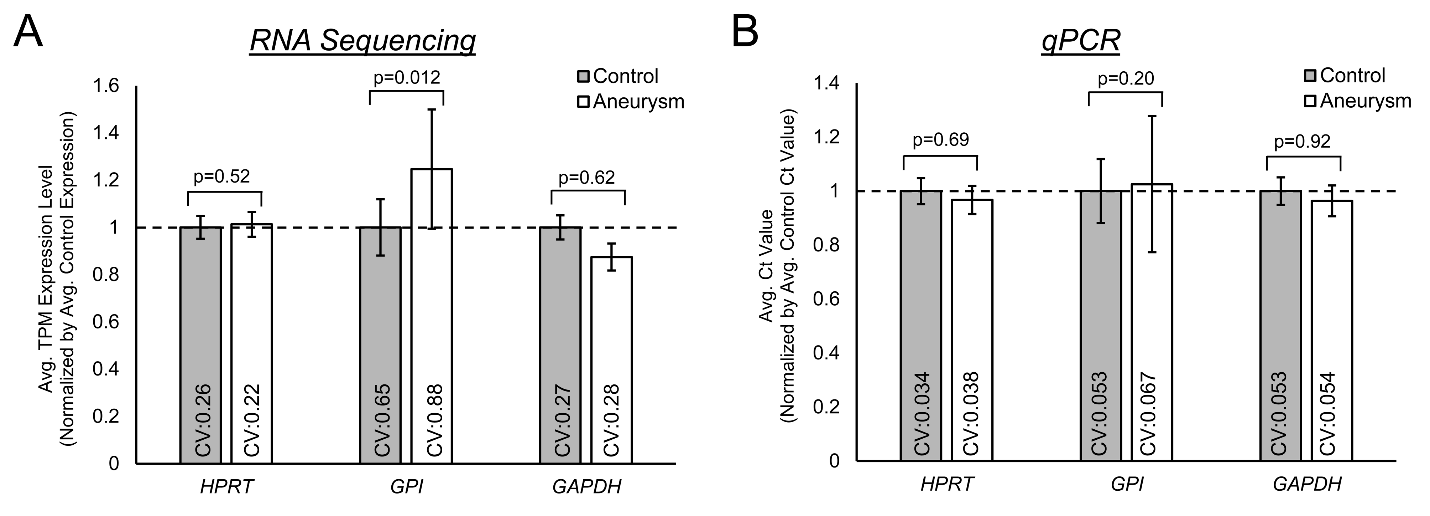
**

**Supplemental Figure 1. Stability of housekeeping genes in RNA sequencing and qPCR.** Variation between control and aneurysm in TPM normalized expression levels from RNA sequencing and C_t_ values from qPCR for each housekeeping gene across all samples on which qPCR was performed were compared by an F-test (significance was defined as p<0.01). We also report their coefficient of variation in the control and aneurysm groups. A). Based on RNA sequencing data, there was no significant difference across the housekeeping genes between the control and aneurysm groups. All coefficients of variation were low (<0.9) and comparable between control and aneurysm. B). Based on qPCR data, there was also no significant difference across the housekeeping genes between the control and aneurysm groups. All coefficients of variation were also low (<0.07) and comparable between control and aneurysm. For all graphs, error bars represent standard error. (CV=coefficient of variation)
