# Supplementary material for: Classification models using circulating neutrophil transcripts can detect unruptured intracranial aneurysm
Source: J Transl Med. 2020 Oct 15;18:392. doi: 10.1186/s12967-020-02550-2 (PMC7565814; doi:10.1186/s12967-020-02550-2)
Supplement: Supplementary file 3 — Additional file 3: Table S2. Characteristics of 73 aneurysms in all patients with intracranial aneurysms. [file 12967_2020_2550_MOESM3_ESM.docx]

**Supplemental Table 2**. **Characteristics of 73 aneurysms in all patients with intracranial aneurysms (12 patients had multiple intracranial aneurysms).***

| **ID** | **Size (mm)** | **Location** | **Additional IAs** | **Fam. Hx.** | **Indication for DSA** |
| --- | --- | --- | --- | --- | --- |
| ***Training cohort*** | |  |  |  |  |
| A1 | 10 | VB junction | No | No | MRI for hand numbness indicated possible IA |
| A2 | 8 | L ICA Paraopthalmic | No | No | Follow-up imaging of known IA |
| A3 | 5.5 | L ICA hypop. | No | No | Not reported |
| A4 | 8 | L superior hypop. | No | No | Not reported |
| A5 | 4.5 | L MCA bif. | No | No | CT for dizziness showed possible IA |
| A6 | 4 | L Ophthalmic | Yes +2 (1.5 mm R ICA, 3 mm clinoid) | No | MRI for headache indicated possible IA |
| A7 | 10.8 | L MCA bif. | Yes +1 (2.28 mm R MCA) | No | Incidental finding on MRI |
| A8 | 9 | R Pcom | No | No | Follow-up imaging of known IA |
| A9 | 3 | BT | No | No | MRI for headache indicated possible IA |
| A10 | 13 | L ACA (A1) | No | Yes | MRI for visual loss MRI indicated possible IA |
| A11 | 19 | L ICA | No | No | MRI for double vision indicated possible IA |
| A12 | 3.2 | R MCA bif. | No | No | CTA imaging indicated possible IA |
| A13 | 5 | L ICA | Yes +1 (2 mm paraclinoid) | No | Not reported |
| A14 | 2.9 | R SCA | No | No | Not reported |
| A15 | 1 | L ICA | No | Yes | Follow-up imaging of known IA |
| A16 | 5 | R supraclinoid | Yes +1 (3.5 mm L para-opthalmic) | Yes | MRI for tremors indicated possible IA |
| A17 | 5.1 | L ICA | No | No | MRI for headache indicated possible IA |
| A18 | 4.85 | R ICA | No | No | CT for headache indicated need for DSA |
| A19 | 3.7 | ACOM | No | No | CTA for headache showed possible IA |
| A20 | 1.4 | R MCA | No | No | Incidental finding on MRI indicated possible IA |
| A21 | 2.8 | L ICA | No | No | MRI for migraine indicated possible IA |
| A22 | 2 | ACA | No | No | Follow-up imaging of known IA |
| A23 | 2 | L ICA | No | No | MRI for migraine indicated possible IA |
| A24 | 3.9 | BT | No | No | MRA for headache indicated possible IA |
| A25 | 2.8 | ICA | No | No | Follow-up imaging of known IA |
| A26 | 3 | R ACA (A1) | Yes +1 (2.38 mm BT) | No | Screening MRA showed basilar aneurysm and A1 aneurysms |
| A27 | 3 | R PCOM | No | Yes | Screening CTA showed PCOM aneurysm |
| A28 | 3 | Cavernous carotid | No | No | Imaging for s symptomatic IA |
| A29 | 8 | R MCA | No | No | Follow-up imaging of known IA |
| A30 | 5 | PCOM | No | Yes | IA discovered on MRA |
| A31 | 2 | MCA | No | No | MRA showed possible IA |
| A32 | 3 | L MCA bif. | Yes +2 (3 AComm, 3 R ophthalmic) | No | noninvasive imaging for vertigo suggested IA |
| A33 | 5 | L PCOM | No | No | IA found on workup for dizziness and weakness |
| A34 | 3 | R cavernous | Yes +1 (2, R petrous ICA) | No | IAs found on imaging for neck pain |
| A35 | 3 | L PCOM | No | No | Imaging for possible infundibulum |
| A36 | 3.4 | R PCOM | Yes +3 (2.4 mm L PCA, 1 mm L sup. Hypop., 2.1 mm R MCA bif.) | No | maxillofacial CT showed incidental IA |
| A37 | 2 | L ICA | No | No | Incidental finding on workup for potential SAH |
| A38 | 7.5 | R MCA | No | No | workup for concussion after car crash |
| A39 | 5.3 | R ICA term. | No | No | MRA for hearing loss suggests IA |
| ***Testing Cohort*** | |  |  |  |  |
| A40 | 5 | BT | No | No | MRA and CT for tremor revealed possible IA |
| A41 | 2 | L MCA (M1) | No | No | Not reported |
| A42 | 6 | R MCA | No | No | MRA for motor vehicle accident indicated IA |
| A43 | 7 | L MCA | Yes +1 (3.5 mm R ACA A1-2 junction) | No | Follow-up imaging of known IA |
| A44 | 10 | L ICA (Paraclinoid) | Yes +2 (9 mm R ICA paraclin., 2 mm R ICA proximal to PCom) | No | MRI/MRA for vision loss showed IA |
| A45 | 5.4 | R Pcom | Yes +1 (L Pcom > 5 mm) | No | Follow-up imaging of known IA |
| A46 | 15 | R ICA | No | No | Workup for WHOL found IA |
| A47 | 4 | R ICA | No | No | 4.3 mm right ICA aneurysm (left CN VI palsy) |
| A48 | 5 | L ICA | No | No | Follow-up imaging of known IA |
| A49 | 3.6 | Cavernous ICA | No | No | MRI for diplopia, ptosis, and neuromyotonia showed IA |
| A50 | 3 | L ICA | Yes +1 (3 mm R Pcom) | Yes | Imaging due to family history |
| A51 | 1 | R SHA | Yes +1 (1 mm L ICA cavernous) | No | CTA indicated possible IA |
| A52 | 4 | R ICA | No | Yes | Follow-up imaging of known IA |
| A53 | 10 | R PCA (fusiform) | No | No | IA found on CTA for dizziness |
| A54 | 3 | R ICA | No | Yes | IA found on work-up for migraines |
| A55 | 5.6 | R ICA | No | No | Not reported |

*Intracranial aneurysm (IA) size ranged from 1 mm to 19 mm in greatest diameter. 30 IAs (55%) were classified as small (diameter <5 mm), and 25 (45%) were classified as large (diameter ≥5 mm). The aneurysms were situated at various locations in the Circle of Willis, with most around the internal carotid artery (ICA) and its branches. Seven patients with IA had a family history of the disease (13%), and 12 patients with IA had multiple IAs (22%). In general, digital subtraction angiography was performed for confirmation of IA presence after an incidental finding of IA on noninvasive imaging or for follow-up imaging of a previously detected IA. (ACA=anterior cerebral artery, AComA=anterior communicating artery, BT=basilar terminus, CT=computed tomography, DSA=digital subtraction angiography, IA=intracranial aneurysm, ICA=internal carotid artery, MCA=middle cerebral artery, MRA=magnetic resonance angiography, MRI=magnetic resonance imaging, PComA=posterior communicating artery, VB=vertebrobasilar)
