# Supplementary material for: Classification models using circulating neutrophil transcripts can detect unruptured intracranial aneurysm
Source: J Transl Med. 2020 Oct 15;18:392. doi: 10.1186/s12967-020-02550-2 (PMC7565814; doi:10.1186/s12967-020-02550-2)
Supplement: Supplementary file 4 — Additional file 4: Table S3. Batch Assignment, RNA QC, and sequencing QC for the testing and training cohorts. [file 12967_2020_2550_MOESM4_ESM.docx]

**Supplemental Table 3. Batch Assignment, RNA QC, and sequencing QC for the testing and training cohorts.***

| **Sample ID** | **Class** | **Batch** | **260/280** | **RIN** | **M Seqs** | **Seq Length** | **%GC** | **% Aligned** |
| --- | --- | --- | --- | --- | --- | --- | --- | --- |
| ***Training Cohort*** | |  |  |  |  |  |  |  |
| C1 | Control | 2 | 1.98 | 7.5 | 45.5 | 51 | 48 | 97 |
| C2 | Control | 1 | 2.06 | 7.9 | 39.9 | 51 | 48 | 96 |
| C3 | Control | 1 | 2.07 | 7.0 | 27.0 | 51 | 48 | 95 |
| C4 | Control | 2 | 1.99 | 6.1 | 31.6 | 51 | 48 | 95 |
| C5 | Control | 1 | 2.02 | 7.4 | 59.3 | 51 | 49 | 94 |
| C6 | Control | 1 | 2.05 | 6.6 | 35.9 | 51 | 49 | 93 |
| C7 | Control | 1 | 2.05 | 7.9 | 97.4 | 51 | 50 | 95 |
| C8 | Control | 1 | 1.92 | 6.6 | 44.2 | 51 | 49 | 95 |
| C9 | Control | 1 | 2.08 | 6.2 | 36.2 | 51 | 49 | 94 |
| C10 | Control | 1 | 2.04 | 7.3 | 79.3 | 51 | 49 | 95 |
| C11 | Control | 1 | 2.04 | 6.6 | 66.0 | 51 | 50 | 94 |
| C12 | Control | 1 | 2.03 | 5.9 | 58.5 | 51 | 51 | 94 |
| C13 | Control | 1 | 2.06 | 5.9 | 64.3 | 51 | 51 | 93 |
| C14 | Control | 1 | 2.10 | 5.5 | 36.9 | 51 | 51 | 95 |
| C15 | Control | 2 | 2.05 | 7.7 | 57.6 | 51 | 46 | 96 |
| C16 | Control | 2 | 2.07 | 6.0 | 61.4 | 51 | 49 | 95 |
| C17 | Control | 2 | 2.10 | 6.1 | 47.1 | 51 | 46 | 94 |
| C18 | Control | 2 | 2.09 | 7.0 | 64.9 | 51 | 45 | 94 |
| C19 | Control | 2 | 2.09 | 7.9 | 65.9 | 51 | 47 | 96 |
| C20 | Control | 2 | 2.06 | 9.0 | 79.2 | 51 | 49 | 96 |
| C21 | Control | 1 | 1.86 | 5.0 | 64.6 | 51 | 50 | 93 |
| C22 | Control | 2 | 2.00 | 8.8 | 61.0 | 51 | 47 | 97 |
| C23 | Control | 2 | 2.02 | 4.5 | 27.8 | 51 | 52 | 94 |
| C24 | Control | 2 | 1.98 | 5.1 | 63.5 | 51 | 48 | 96 |
| C25 | Control | 2 | 2.05 | 8.0 | 68.3 | 51 | 47 | 97 |
| C26 | Control | 2 | 2.07 | 5.2 | 62.3 | 51 | 49 | 95 |
| C27 | Control | 2 | 2.06 | 5.3 | 73.1 | 51 | 49 | 95 |
| C28 | Control | 1 | 2.02 | 8.2 | 47.3 | 51 | 50 | 94 |
| C29 | Control | 2 | 2.02 | 6.6 | 63.4 | 51 | 49 | 95 |
| C30 | Control | 2 | 2.01 | 5.5 | 70.9 | 51 | 50 | 97 |
| C31 | Control | 2 | 2.05 | 8.5 | 45.7 | 51 | 47 | 93 |
| C32 | Control | 2 | 2.06 | 6.7 | 55.1 | 51 | 47 | 97 |
| C33 | Control | 2 | 1.96 | 6.1 | 46.2 | 51 | 50 | 94 |
| C34 | Control | 2 | 2.08 | 7.8 | 46.6 | 51 | 46 | 95 |
| C35 | Control | 2 | 2.10 | 8.1 | 64.5 | 51 | 47 | 96 |
| C36 | Control | 2 | 2.08 | 8.4 | 36.1 | 51 | 45 | 94 |
| C37 | Control | 2 | 2.04 | 7.2 | 32.3 | 51 | 47 | 96 |
| C38 | Control | 2 | 2.07 | 6.9 | 46.2 | 51 | 45 | 93 |
| C39 | Control | 2 | 2.08 | 7.9 | 58.0 | 51 | 47 | 96 |
| C40 | Control | 2 | 1.98 | 4.6 | 67.4 | 51 | 52 | 96 |
| C41 | Control | 2 | 2.08 | 6.5 | 60.8 | 51 | 46 | 96 |
| C42 | Control | 2 | 2.04 | 5.2 | 64.3 | 51 | 47 | 95 |
| C43 | Control | 2 | 2.05 | 7.8 | 54.6 | 51 | 47 | 93 |
| C44 | Control | 2 | 2.01 | 6.7 | 65.1 | 51 | 45 | 97 |
| C45 | Control | 2 | 2.01 | 6.7 | 41.9 | 51 | 47 | 96 |
| C46 | Control | 2 | 2.04 | 6.8 | 68.7 | 51 | 47 | 95 |
| C47 | Control | 2 | 1.99 | 4.8 | 50.8 | 51 | 54 | 95 |
| C48 | Control | 2 | 2.09 | 8.0 | 66.2 | 51 | 47 | 96 |
| C49 | Control | 2 | 2.04 | 4.9 | 53.9 | 51 | 46 | 94 |
| C50 | Control | 2 | 2.04 | 7.2 | 55.9 | 51 | 46 | 95 |
| C51 | Control | 2 | 2.06 | 7.4 | 51.8 | 51 | 46 | 93 |
| C52 | Control | 2 | 2.04 | 7.3 | 41.5 | 51 | 47 | 92 |
| C53 | Control | 2 | 2.08 | 6.5 | 78.3 | 51 | 46 | 96 |
| C54 | Control | 2 | 2.06 | 5.1 | 45.0 | 51 | 48 | 95 |
| C55 | Control | 2 | 2.07 | 8.1 | 103.7 | 51 | 48 | 96 |
| A1 | Aneurysm | 1 | 2.04 | 7.8 | 48.1 | 51 | 49 | 96 |
| A2 | Aneurysm | 1 | 2.07 | 7.5 | 35.7 | 51 | 48 | 95 |
| A3 | Aneurysm | 1 | 2.07 | 7.1 | 34.0 | 51 | 49 | 95 |
| A4 | Aneurysm | 2 | 1.93 | 6.0 | 47.6 | 51 | 48 | 94 |
| A5 | Aneurysm | 1 | 2.06 | 8.1 | 60.4 | 51 | 49 | 95 |
| A6 | Aneurysm | 1 | 2.02 | 6.1 | 55.9 | 51 | 49 | 95 |
| A7 | Aneurysm | 1 | 2.03 | 7.3 | 61.3 | 51 | 49 | 95 |
| A8 | Aneurysm | 1 | 1.99 | 6.5 | 23.4 | 51 | 49 | 94 |
| A9 | Aneurysm | 1 | 1.97 | 6.9 | 14.6 | 51 | 49 | 93 |
| A10 | Aneurysm | 1 | 2.05 | 7.7 | 26.9 | 51 | 50 | 96 |
| A11 | Aneurysm | 1 | 2.07 | 6.0 | 29.1 | 51 | 50 | 95 |
| A12 | Aneurysm | 2 | 1.98 | 5.8 | 48.0 | 51 | 50 | 95 |
| A13 | Aneurysm | 2 | 2.04 | 5.6 | 66.5 | 51 | 46 | 95 |
| A14 | Aneurysm | 2 | 2.03 | 5.7 | 36.2 | 51 | 49 | 97 |
| A15 | Aneurysm | 1 | 2.07 | 7.8 | 32.3 | 51 | 50 | 87 |
| A16 | Aneurysm | 1 | 1.95 | 7.2 | 59.5 | 51 | 49 | 94 |
| A17 | Aneurysm | 1 | 2.08 | 7.1 | 55.6 | 51 | 51 | 92 |
| A18 | Aneurysm | 1 | 2.06 | 7.1 | 72.8 | 51 | 48 | 78 |
| A19 | Aneurysm | 1 | 2.00 | 6.9 | 64.9 | 51 | 50 | 95 |
| A20 | Aneurysm | 1 | 1.97 | 6.4 | 75.3 | 51 | 51 | 94 |
| A21 | Aneurysm | 2 | 2.09 | 6.0 | 48.7 | 51 | 47 | 96 |
| A22 | Aneurysm | 1 | 2.06 | 5.9 | 42.2 | 51 | 51 | 94 |
| A23 | Aneurysm | 1 | 2.12 | 7.3 | 47.2 | 51 | 51 | 88 |
| A24 | Aneurysm | 1 | 1.96 | 7.4 | 42.1 | 51 | 50 | 94 |
| A25 | Aneurysm | 2 | 2.02 | 7.6 | 59.3 | 51 | 49 | 97 |
| A26 | Aneurysm | 2 | 2.01 | 5.8 | 79.6 | 51 | 48 | 96 |
| A27 | Aneurysm | 2 | 2.02 | 5.2 | 41.1 | 51 | 48 | 95 |
| A28 | Aneurysm | 2 | 2.03 | 4.6 | 56.2 | 51 | 50 | 96 |
| A29 | Aneurysm | 2 | 2.06 | 7.4 | 58.5 | 51 | 49 | 97 |
| A30 | Aneurysm | 2 | 1.91 | 6.6 | 56.9 | 51 | 48 | 94 |
| A31 | Aneurysm | 2 | 2.06 | 6.0 | 56.4 | 51 | 47 | 95 |
| A32 | Aneurysm | 2 | 1.99 | 6.0 | 56.3 | 51 | 48 | 96 |
| A33 | Aneurysm | 2 | 2.06 | 8.2 | 45.8 | 51 | 46 | 96 |
| A34 | Aneurysm | 2 | 2.07 | 6.1 | 52.4 | 51 | 45 | 94 |
| A35 | Aneurysm | 2 | 2.07 | 7.2 | 53.4 | 51 | 48 | 96 |
| A36 | Aneurysm | 2 | 2.00 | 6.1 | 50.8 | 51 | 49 | 96 |
| A37 | Aneurysm | 2 | 2.07 | 5.7 | 66.3 | 51 | 48 | 95 |
| A38 | Aneurysm | 2 | 2.06 | 5.3 | 72.7 | 51 | 52 | 96 |
| A39 | Aneurysm | 2 | 2.10 | 5.1 | 19.9 | 51 | 48 | 96 |
| ***Testing Cohort*** | |  |  |  |  |  |  |  |
| C56 | Control | 1 | 2.07 | 7.3 | 21.1 | 51 | 49 | 95 |
| C57 | Control | 1 | 2.06 | 6.3 | 41.9 | 51 | 49 | 91 |
| C58 | Control | 1 | 1.92 | 6.5 | 68.9 | 51 | 49 | 95 |
| C59 | Control | 1 | 1.99 | 7.1 | 53.3 | 51 | 50 | 94 |
| C60 | Control | 1 | 2.08 | 7.1 | 80.3 | 51 | 50 | 95 |
| C61 | Control | 1 | 1.96 | 6.7 | 67.8 | 51 | 49 | 95 |
| C62 | Control | 1 | 1.97 | 6.4 | 89.1 | 51 | 50 | 94 |
| C63 | Control | 1 | 2.05 | 6.0 | 74.4 | 51 | 50 | 94 |
| C64 | Control | 1 | 2.01 | 7.9 | 51.5 | 51 | 50 | 94 |
| C65 | Control | 2 | 2.09 | 9.0 | 51.5 | 51 | 46 | 97 |
| C66 | Control | 2 | 2.00 | 5.9 | 53.8 | 51 | 49 | 95 |
| C67 | Control | 2 | 2.08 | 9.1 | 66.9 | 51 | 48 | 97 |
| C68 | Control | 2 | 2.02 | 5.3 | 57.3 | 51 | 51 | 95 |
| C69 | Control | 2 | 2.05 | 6.2 | 82.8 | 51 | 48 | 97 |
| C70 | Control | 2 | 2.06 | 8.9 | 61.6 | 51 | 48 | 96 |
| C71 | Control | 2 | 2.08 | 5.9 | 91.6 | 51 | 47 | 97 |
| C72 | Control | 2 | 2.08 | 5.7 | 54.8 | 51 | 46 | 95 |
| C73 | Control | 2 | 2.08 | 7.6 | 63.2 | 51 | 46 | 90 |
| C74 | Control | 2 | 2.08 | 6.7 | 50.1 | 51 | 46 | 96 |
| C75 | Control | 2 | 2.09 | 6.8 | 43.9 | 51 | 47 | 95 |
| C76 | Control | 2 | 2.01 | 6.5 | 58.7 | 51 | 46 | 94 |
| C77 | Control | 2 | 2.07 | 5.4 | 52.4 | 51 | 47 | 93 |
| C78 | Control | 2 | 2.04 | 7.7 | 53.9 | 51 | 49 | 96 |
| C79 | Control | 2 | 1.87 | 5.2 | 55.6 | 51 | 49 | 94 |
| A40 | Aneurysm | 1 | 2.02 | 7.5 | 35.8 | 51 | 49 | 95 |
| A41 | Aneurysm | 2 | 2.03 | 5.7 | 27.1 | 51 | 49 | 94 |
| A42 | Aneurysm | 1 | 2.07 | 6.7 | 86.9 | 51 | 51 | 83 |
| A43 | Aneurysm | 1 | 2.03 | 7.2 | 39.8 | 51 | 50 | 96 |
| A44 | Aneurysm | 2 | 2.06 | 8.1 | 82.7 | 51 | 48 | 97 |
| A45 | Aneurysm | 2 | 2.05 | 5.1 | 48.4 | 51 | 48 | 96 |
| A46 | Aneurysm | 2 | 2.04 | 5.1 | 56.9 | 51 | 49 | 96 |
| A47 | Aneurysm | 2 | 2.06 | 5.1 | 53.6 | 51 | 48 | 96 |
| A48 | Aneurysm | 2 | 2.08 | 7.9 | 29.3 | 51 | 47 | 95 |
| A49 | Aneurysm | 2 | 2.06 | 7.9 | 53.2 | 51 | 47 | 96 |
| A50 | Aneurysm | 2 | 1.76 | 4.8 | 33.7 | 51 | 48 | 90 |
| A51 | Aneurysm | 2 | 2.03 | 4.8 | 66.1 | 51 | 47 | 94 |
| A52 | Aneurysm | 2 | 1.90 | 4.5 | 39.0 | 51 | 53 | 95 |
| A53 | Aneurysm | 2 | 2.07 | 8.2 | 82.3 | 51 | 48 | 96 |
| A54 | Aneurysm | 2 | 2.08 | 5.4 | 37.0 | 51 | 50 | 95 |
| A55 | Aneurysm | 2 | 2.10 | 7.7 | 104.8 | 51 | 49 | 96 |

*Over the study period, samples were collected and sequenced in 2 main batches. The earlier batch is designated “1,” and the later batch is designated “2.” When creating the training and testing cohorts, data from each batch were randomly partitioned into each group. The quality of the RNA samples was assessed by the 260/280 ratio and the RIN. QC of sequencing showed that prior to alignment, all samples had an average of 53.84 M sequences. The sequencing experiments had an average of 48.4 M mapped reads with a 95.37% read mapping rate and detected an average of 11,591 transcripts (transcripts with TPM>1 after batch effect correction). (M.=million, Qual.=quality, Seqs.=sequences, TPM=transcripts per million, QC=quality control, RIN=RNA integrity number)
