# Supplementary material for: Classification models using circulating neutrophil transcripts can detect unruptured intracranial aneurysm
Source: J Transl Med. 2020 Oct 15;18:392. doi: 10.1186/s12967-020-02550-2 (PMC7565814; doi:10.1186/s12967-020-02550-2)
Supplement: Supplementary file 5 — Additional file 5: Figure S2. Co-variate correlation analysis between RNA quality (RIN) and differentially expressed genes. [file 12967_2020_2550_MOESM5_ESM.docx]

**
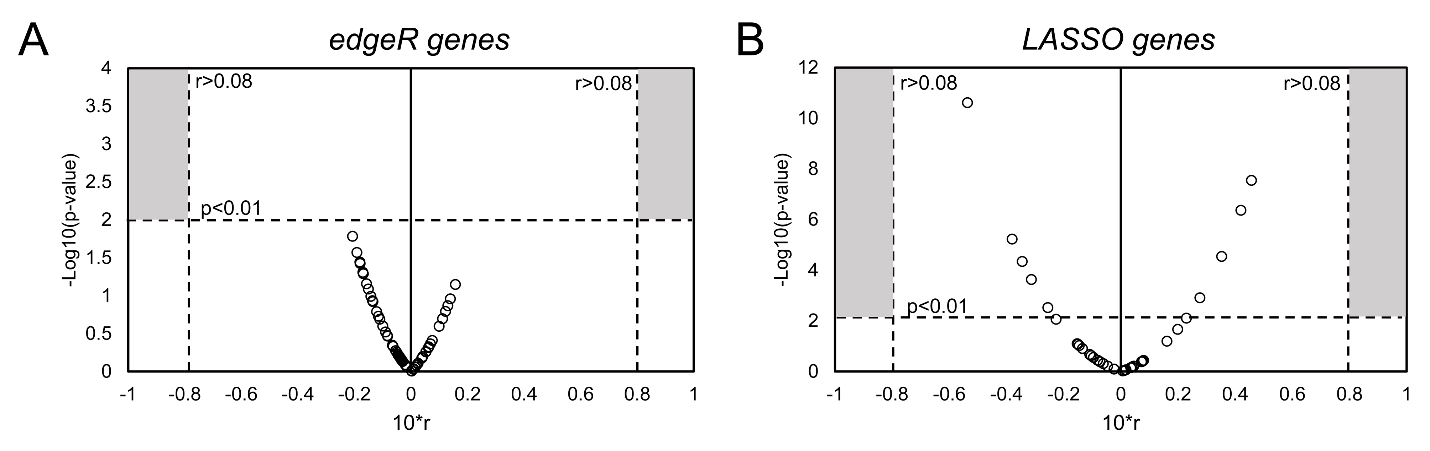
**

**Supplemental Figure 2. Co-variate correlation analysis between RNA quality (RIN) and differentially expressed genes.** For each gene of interest, the -Log10(p-value) is plotted against the Pearson correlation coefficients (r) multiplied by 10. Significant correlations in expression level and RIN as a co-variate, defined as abs(r)>0.80 and p<0.01 would appear in the top left and right corners of each plot (in grey). A). There were no significant correlations for genes identified as significant by edgeR analysis. B). There were also no significant correlations for genes selected for model training by LASSO.
