# Supplementary material for: Classification models using circulating neutrophil transcripts can detect unruptured intracranial aneurysm
Source: J Transl Med. 2020 Oct 15;18:392. doi: 10.1186/s12967-020-02550-2 (PMC7565814; doi:10.1186/s12967-020-02550-2)
Supplement: Supplementary file 6 — Additional file 6: Table S4. GORILLA enriched ontologies for edgeR genes. [file 12967_2020_2550_MOESM6_ESM.docx]

**Supplemental Table 4. GORILLA enriched ontologies for edgeR genes.**

| **Gene Set** | **Type** | **GO Term** | **Description** | **P-value** | **Enrichment** |
| --- | --- | --- | --- | --- | --- |
| **Genes with lower expression in IA** | Process | [GO:0099505](http://www.godatabase.org/cgi-bin/amigo/go.cgi?query=GO:0099505&view=details) | regulation of presynaptic membrane potential | 0.000641 | 1559.29 |
|  | Process | [GO:0061743](http://www.godatabase.org/cgi-bin/amigo/go.cgi?query=GO:0061743&view=details) | motor learning | 0.000641 | 1559.29 |
|  | Process | [GO:0098912](http://www.godatabase.org/cgi-bin/amigo/go.cgi?query=GO:0098912&view=details) | membrane depolarization during atrial cardiac muscle cell action potential | 0.000641 | 1559.29 |
|  | Process | [GO:0003026](http://www.godatabase.org/cgi-bin/amigo/go.cgi?query=GO:0003026&view=details) | regulation of systemic arterial blood pressure by aortic arch baroreceptor feedback | 0.000641 | 1559.29 |
|  | Process | [GO:0086067](http://www.godatabase.org/cgi-bin/amigo/go.cgi?query=GO:0086067&view=details) | AV node cell to bundle of His cell communication | 0.000641 | 1559.29 |
|  | Process | [GO:0086015](http://www.godatabase.org/cgi-bin/amigo/go.cgi?query=GO:0086015&view=details) | SA node cell action potential | 0.000641 | 1559.29 |
|  | Process | [GO:0086016](http://www.godatabase.org/cgi-bin/amigo/go.cgi?query=GO:0086016&view=details) | AV node cell action potential | 0.000641 | 1559.29 |
|  | Process | [GO:0086048](http://www.godatabase.org/cgi-bin/amigo/go.cgi?query=GO:0086048&view=details) | membrane depolarization during bundle of His cell action potential | 0.000641 | 1559.29 |
|  | Process | [GO:0086045](http://www.godatabase.org/cgi-bin/amigo/go.cgi?query=GO:0086045&view=details) | membrane depolarization during AV node cell action potential | 0.000641 | 1559.29 |
|  | Process | [GO:0086043](http://www.godatabase.org/cgi-bin/amigo/go.cgi?query=GO:0086043&view=details) | bundle of His cell action potential | 0.000641 | 1559.29 |
|  | Process | [GO:0050915](http://www.godatabase.org/cgi-bin/amigo/go.cgi?query=GO:0050915&view=details) | sensory perception of sour taste | 0.000641 | 1559.29 |
|  | Process | [GO:0007196](http://www.godatabase.org/cgi-bin/amigo/go.cgi?query=GO:0007196&view=details) | adenylate cyclase-inhibiting G protein-coupled glutamate receptor signaling pathway | 0.000641 | 1559.29 |
|  | Process | [GO:0035725](http://www.godatabase.org/cgi-bin/amigo/go.cgi?query=GO:0035725&view=details) | sodium ion transmembrane transport | 0.000142 | 107.54 |
|  | Process | [GO:0007417](http://www.godatabase.org/cgi-bin/amigo/go.cgi?query=GO:0007417&view=details) | central nervous system development | 0.000409 | 63.64 |
|  | Process | [GO:0006814](http://www.godatabase.org/cgi-bin/amigo/go.cgi?query=GO:0006814&view=details) | sodium ion transport | 0.000497 | 57.75 |
|  | Process | [GO:0098916](http://www.godatabase.org/cgi-bin/amigo/go.cgi?query=GO:0098916&view=details) | anterograde trans-synaptic signaling | 0.000957 | 41.58 |
|  | Process | [GO:0007268](http://www.godatabase.org/cgi-bin/amigo/go.cgi?query=GO:0007268&view=details) | chemical synaptic transmission | 0.000957 | 41.58 |
|  | Process | [GO:0042391](http://www.godatabase.org/cgi-bin/amigo/go.cgi?query=GO:0042391&view=details) | regulation of membrane potential | 0.000118 | 28.01 |
|  | Process | [GO:0023052](http://www.godatabase.org/cgi-bin/amigo/go.cgi?query=GO:0023052&view=details) | signaling | 0.000184 | 24.11 |
|  | Function | [GO:0099507](http://www.godatabase.org/cgi-bin/amigo/go.cgi?query=GO:0099507&view=details) | ligand-gated ion channel activity involved in regulation of presynaptic membrane potential | 6.41E-04 | 1559.29 |
|  | Function | [GO:0001640](http://www.godatabase.org/cgi-bin/amigo/go.cgi?query=GO:0001640&view=details) | adenylate cyclase inhibiting G protein-coupled glutamate receptor activity | 6.41E-04 | 1559.29 |
|  | Function | [GO:0098988](http://www.godatabase.org/cgi-bin/amigo/go.cgi?query=GO:0098988&view=details) | G protein-coupled glutamate receptor activity | 6.41E-04 | 1559.29 |
|  | Function | [GO:0015277](http://www.godatabase.org/cgi-bin/amigo/go.cgi?query=GO:0015277&view=details) | kainate selective glutamate receptor activity | 6.41E-04 | 1559.29 |
|  | Function | [GO:0086060](http://www.godatabase.org/cgi-bin/amigo/go.cgi?query=GO:0086060&view=details) | voltage-gated sodium channel activity involved in AV node cell action potential | 6.41E-04 | 1559.29 |
|  | Function | [GO:0086061](http://www.godatabase.org/cgi-bin/amigo/go.cgi?query=GO:0086061&view=details) | voltage-gated sodium channel activity involved in bundle of His cell action potential | 6.41E-04 | 1559.29 |
|  | Function | [GO:0086063](http://www.godatabase.org/cgi-bin/amigo/go.cgi?query=GO:0086063&view=details) | voltage-gated sodium channel activity involved in SA node cell action potential | 6.41E-04 | 1559.29 |
|  | Function | [GO:0005272](http://www.godatabase.org/cgi-bin/amigo/go.cgi?query=GO:0005272&view=details) | sodium channel activity | 9.03E-09 | 584.73 |
|  | Function | [GO:0099094](http://www.godatabase.org/cgi-bin/amigo/go.cgi?query=GO:0099094&view=details) | ligand-gated cation channel activity | 1.32E-04 | 111.38 |
|  | Function | [GO:0015081](http://www.godatabase.org/cgi-bin/amigo/go.cgi?query=GO:0015081&view=details) | sodium ion transmembrane transporter activity | 2.12E-06 | 106.31 |
|  | Function | [GO:0022834](http://www.godatabase.org/cgi-bin/amigo/go.cgi?query=GO:0022834&view=details) | ligand-gated channel activity | 1.52E-04 | 103.95 |
|  | Function | [GO:0015276](http://www.godatabase.org/cgi-bin/amigo/go.cgi?query=GO:0015276&view=details) | ligand-gated ion channel activity | 1.52E-04 | 103.95 |
|  | Function | [GO:0022839](http://www.godatabase.org/cgi-bin/amigo/go.cgi?query=GO:0022839&view=details) | ion gated channel activity | 1.92E-05 | 51.41 |
|  | Function | [GO:0005261](http://www.godatabase.org/cgi-bin/amigo/go.cgi?query=GO:0005261&view=details) | cation channel activity | 2.04E-05 | 50.3 |
|  | Function | [GO:0022836](http://www.godatabase.org/cgi-bin/amigo/go.cgi?query=GO:0022836&view=details) | gated channel activity | 2.18E-05 | 49.24 |
|  | Function | [GO:0005216](http://www.godatabase.org/cgi-bin/amigo/go.cgi?query=GO:0005216&view=details) | ion channel activity | 5.08E-05 | 37.13 |
|  | Function | [GO:0022838](http://www.godatabase.org/cgi-bin/amigo/go.cgi?query=GO:0022838&view=details) | substrate-specific channel activity | 5.45E-05 | 36.26 |
|  | Function | [GO:0022803](http://www.godatabase.org/cgi-bin/amigo/go.cgi?query=GO:0022803&view=details) | passive transmembrane transporter activity | 6.53E-05 | 34.14 |
|  | Function | [GO:0015267](http://www.godatabase.org/cgi-bin/amigo/go.cgi?query=GO:0015267&view=details) | channel activity | 6.53E-05 | 34.14 |
|  | Function | [GO:0015077](http://www.godatabase.org/cgi-bin/amigo/go.cgi?query=GO:0015077&view=details) | monovalent inorganic cation transmembrane transporter activity | 8.55E-05 | 31.19 |
|  | Function | [GO:0046873](http://www.godatabase.org/cgi-bin/amigo/go.cgi?query=GO:0046873&view=details) | metal ion transmembrane transporter activity | 1.02E-04 | 29.42 |
|  | Function | [GO:0022890](http://www.godatabase.org/cgi-bin/amigo/go.cgi?query=GO:0022890&view=details) | inorganic cation transmembrane transporter activity | 3.61E-04 | 19.17 |
|  | Function | [GO:0008324](http://www.godatabase.org/cgi-bin/amigo/go.cgi?query=GO:0008324&view=details) | cation transmembrane transporter activity | 4.97E-04 | 17.2 |
|  | Function | [GO:0015318](http://www.godatabase.org/cgi-bin/amigo/go.cgi?query=GO:0015318&view=details) | inorganic molecular entity transmembrane transporter activity | 9.97E-04 | 13.56 |
| **Genes with higher expression in IA** | Process | [GO:0021797](http://www.godatabase.org/cgi-bin/amigo/go.cgi?query=GO:0021797&view=details) | forebrain anterior/posterior pattern specification | 1.76E-06 | 728.07 |
|  | Process | [GO:0022029](http://www.godatabase.org/cgi-bin/amigo/go.cgi?query=GO:0022029&view=details) | telencephalon cell migration | 2.37E-04 | 85.65 |
|  | Process | [GO:0021885](http://www.godatabase.org/cgi-bin/amigo/go.cgi?query=GO:0021885&view=details) | forebrain cell migration | 2.37E-04 | 85.65 |
|  | Process | [GO:0072678](http://www.godatabase.org/cgi-bin/amigo/go.cgi?query=GO:0072678&view=details) | T cell migration | 3.64E-04 | 69.34 |
|  | Process | [GO:0044364](http://www.godatabase.org/cgi-bin/amigo/go.cgi?query=GO:0044364&view=details) | disruption of cells of other organism | 4.78E-04 | 60.67 |
|  | Process | [GO:0031640](http://www.godatabase.org/cgi-bin/amigo/go.cgi?query=GO:0031640&view=details) | killing of cells of other organism | 4.78E-04 | 60.67 |
|  | Process | [GO:0061844](http://www.godatabase.org/cgi-bin/amigo/go.cgi?query=GO:0061844&view=details) | antimicrobial humoral immune response mediated by antimicrobial peptide | 9.07E-04 | 44.13 |
|  | Process | [GO:0072676](http://www.godatabase.org/cgi-bin/amigo/go.cgi?query=GO:0072676&view=details) | lymphocyte migration | 9.63E-04 | 42.83 |
|  | Process | [GO:0035821](http://www.godatabase.org/cgi-bin/amigo/go.cgi?query=GO:0035821&view=details) | modification of morphology or physiology of other organism | 3.41E-04 | 21.21 |
|  | Process | [GO:0016477](http://www.godatabase.org/cgi-bin/amigo/go.cgi?query=GO:0016477&view=details) | cell migration | 1.96E-04 | 8.51 |
|  | Process | [GO:0048870](http://www.godatabase.org/cgi-bin/amigo/go.cgi?query=GO:0048870&view=details) | cell motility | 3.12E-04 | 7.7 |
|  | Process | [GO:0040011](http://www.godatabase.org/cgi-bin/amigo/go.cgi?query=GO:0040011&view=details) | locomotion | 5.29E-04 | 6.87 |
