# Supplementary material for: Classification models using circulating neutrophil transcripts can detect unruptured intracranial aneurysm
Source: J Transl Med. 2020 Oct 15;18:392. doi: 10.1186/s12967-020-02550-2 (PMC7565814; doi:10.1186/s12967-020-02550-2)
Supplement: Supplementary file 7 — Additional file 7: Table S5. Transcripts and functions for the significant networks constructed by Ingenuity Pathway Analysis (IPA) using genes identified by edgeR. [file 12967_2020_2550_MOESM7_ESM.docx]

**Supplemental Table 5. Transcripts and functions for the significant networks constructed by Ingenuity Pathway Analysis (IPA) using genes identified by edgeR.***

| **Network** | **Molecules in Network** | **P-score** | **Focus Molecules** | **Top Diseases and Functions** |
| --- | --- | --- | --- | --- |
| **1** | 5-hydroxytryptamine, APP, ASIC1, **ASIC2**, ASIC3, BSND, CDK5R2, **DLGAP2** **[ERICH1-AS1]**, **FCRL4**, **FEZF2**, FYN, GRI, GRIK1, **GRIK3**, GRIK4, GRIK5, GRIN2A, GRIN2D, Grik, IRS1, KCNA4, KCNJ1, L-glutamic acid, LDL, **LINC00599**, LY9, PLEKHA1, **POU3F3**, PTPN11, SLC17A7, **SNTG1**, **TBC1D3** (includes others), TSPOAP1, glutamate receptor, phosphatidylinositol-3,4,5-triphosphate | 21 | 9 | Cell-To-Cell Signaling and Interaction, Nervous System Development and Function, Cell Morphology |
| **2** | ACKR3, ADAMTS4, ADRB2, BAG3, BIRC5, C12orf75, **C1QL1**, CBLN4, CCND1, COL10A1, **COL11A1**, COL9A1, CRHR2, **CTAG2**, ELAVL1, F7, FBXO4, **FMOD**, FMR1, KRT1, KRT10, **KRT9**, LUM, **NPIPA8** (includes others), NPTX1, **PDE9A**, RNF31, **RPL39L**, **SIX3**, SRPK2, TAF4, TLE4, WBP2, YKT6, ZFHX3 | 21 | 9 | Dermatological Diseases and Conditions, Organismal Injury and Abnormalities, Connective Tissue Development and Function |
| **3** | ACKR3, **ADGRG7**, C1q, CACNA2D2, CCR4, CCR6, CFH, CNPY3, COCH, CPD, CRHR2, CTSF, CXCR6, Ca2+, **DEFA1** (includes others), **GPR15**, Gpcr, HTN3, **IFI27**, KCNJ2, LTB4R, MBL2, **MUC7**, NRTN, RCAN2, SCN1B, **SCN5A**, SIGMAR1, SLAMF6, SLC12A1, STATH, TMEM176B, TNF, **ZBTB16**, voltage-gated sodium channel | 15 | 7 | Cell Death and Survival, Connective Tissue Disorders, Inflammatory Disease |

*Transcripts in bold are part of the LASSO gene panel
