# Supplementary material for: Classification models using circulating neutrophil transcripts can detect unruptured intracranial aneurysm
Source: J Transl Med. 2020 Oct 15;18:392. doi: 10.1186/s12967-020-02550-2 (PMC7565814; doi:10.1186/s12967-020-02550-2)
Supplement: Supplementary file 8 — Additional file 8: Table S6. GORILLA enriched ontologies for LASSO genes. [file 12967_2020_2550_MOESM8_ESM.docx]

**Supplemental Table 6. GORILLA enriched ontologies for LASSO genes.***

| **Gene Set** | **Type** | **GO Term** | **Description** | **P-value** | **Enrichment** |
| --- | --- | --- | --- | --- | --- |
| **Genes with higher expression in IA** | N/A | N/A | N/A | N/A | N/A |
| **Genes with lower expression in IA** | Process | GO:1900118 | negative regulation of execution phase of apoptosis | 1.42E-04 | 109.14 |
|  | Process | GO:0001937 | negative regulation of endothelial cell proliferation | 4.80E-04 | 60.63 |
|  | Process | GO:1900117 | regulation of execution phase of apoptosis | 6.56E-04 | 51.97 |

*No ontologies were associated with LASSO genes with higher expression in IA
