# Supplementary material for: Classification models using circulating neutrophil transcripts can detect unruptured intracranial aneurysm
Source: J Transl Med. 2020 Oct 15;18:392. doi: 10.1186/s12967-020-02550-2 (PMC7565814; doi:10.1186/s12967-020-02550-2)
Supplement: Supplementary file 9 — Additional file 9: Table S7. Transcripts and functions for the significant networks constructed by Ingenuity Pathway Analysis (IPA) using genes identified by LASSO. [file 12967_2020_2550_MOESM9_ESM.docx]

**Supplemental Table 7. Transcripts and functions for the significant networks constructed by Ingenuity Pathway Analysis (IPA) using genes identified by LASSO.***

| **Network** | **Molecules in Network** | **P-score** | **Focus Molecules** | **Top Diseases and Functions** |
| --- | --- | --- | --- | --- |
| **1** | 14-3-3, ACTB, AURKB, **C1QL1**, C1QTNF1, CD1E, CD44, **CERS4**, **CLP1**, CUL3, **DCUN1D1**, **EIF4EBP3**, FGF2, FUT3, **GPR15**, **GPR157**, **HIST1H4E**, HSP90AA1, **ISY1**, MMP3, **MORC3**, MPRIP, **MTRNR2L1**, **NECAB1**, **NEIL3**, P2RX, **RFFL**, SDC4, **SDCBP2**, **SYP**, TNF,  TP53 TUBA1C, **USF1**, **UTY** | 47 | 18 | Cancer, Cellular Movement, Connective Tissue Disorder |
| **2** | Basal transcriptional machinery, **CEP295NL**, CIP2A, COIL, CREB1,  **FLT1**, **GTF2B**, H1F0, **HBB**, HBE1, HIST1H1T, HIST1H2BA, HIST1H2BH, HIST1H2BJ, **HIST2H2AB**, **KIAA1324**, **KIAA1614**, KLF7, **LRRN3**, Mapk, MCM5, NGF, NSD1, **PDCD10**, POLB, RECQL, RNA polymerase II, ROBO4, SPAST, SSRP1,  SVIL, **TGS1**, **TMC4**, TMCO4, TMEM245 | 25 | 11 | Cell Cycle, Cellular Assembly and Organization, DNA Replication, Recombination, and Repair |

*Transcripts in bold are part of the LASSO gene panel
